# Supplementary material for: True Prevalence of Unforeseen N2 Disease in NSCLC: A Systematic Review + Meta-Analysis
Source: Cancers (Basel). 2023 Jul 3;15(13):3475. doi: 10.3390/cancers15133475 (PMC10340342; doi:10.3390/cancers15133475)
Supplement: Supplementary file 1 [file cancers-15-03475-s001.zip › cancers-2402097-File S1.pdf]

Excluded papers based on inappropriate study design or cohort

Abe J, Matsumura Y, Shiono S, Aoki M, Sato M, Oura H, Kato H, Minowa M, Oizumi H, Sagawa M, Sakurada A, Okada Y. Validation of the Proposed cN2 Subclassification in the Eighth Edition of the IASLC Staging System: A Prospective Phase II Multicenter Study. JTO Clin Res Rep. 2020 Feb 27;1(2):100019. doi: 10.1016/j.jtocrr.2020.100019. PMID: 34589926; PMCID: PMC8474189.

Adachi H, Sakamaki K, Nishii T, Yamamoto T, Nagashima T, Ishikawa Y, Ando K, Yamanaka K, Watanabe K, Kumakiri Y, Tsuboi M, Maehara T, Nakayama H, Masuda M. Lobe-Specific Lymph Node Dissection as a Standard Procedure in Surgery for Non-Small Cell Lung Cancer: A Propensity Score Matching Study. J Thorac Oncol. 2017 Jan;12(1):85-93. doi: 10.1016/j.jtho.2016.08.127. Epub 2016 Aug 20. PMID: 27553515.

Adams K, Shah PL, Edmonds L, Lim E. Test performance of endobronchial ultrasound and transbronchial needle aspiration biopsy for mediastinal staging in patients with lung cancer: systematic review and meta-analysis. Thorax. 2009 Sep;64(9):757-62. doi: 10.1136/thx.2008.109868. Epub 2009 May 18. PMID: 19454408.

Albes JM, Lietzenmayer R, Schott U, Schülen E, Wehrmann M, Ziemer G. Improvement of non-small-cell lung cancer staging by means of positron emission tomography. Thorac Cardiovasc Surg. 1999 Feb;47(1):42-7. doi: 10.1055/s-2007-1013107. PMID: 10218620.

Al-Ibraheem A, Hirmas N, Fanti S, Paez D, Abuhijla F, Al-Rimawi D, Al-Rasheed U, Abdeljalil R, Hawari F, Alrabi K, Mansour A. Impact of 18F-FDG PET/CT, CT and EBUS/TBNA on preoperative mediastinal nodal staging of NSCLC. BMC Med Imaging. 2021 Mar 17;21(1):49. doi: 10.1186/s12880-021-00580-w. PMID: 33731050; PMCID: PMC7967993.

Altorki N, Wang X, Kozono D, Watt C, Landrenau R, Wigle D, Port J, Jones DR, Conti M, Ashrafi AS, Liberman M, Yasufuku K, Yang S, Mitchell JD, Pass H, Keenan R, Bauer T, Miller D, Kohman LJ, Stinchcombe TE, Vokes E. Lobar or Sublobar Resection for Peripheral Stage IA Non-Small-Cell Lung Cancer. N Engl J Med. 2023 Feb 9;388(6):489-498. doi: 10.1056/NEJMoa2212083. PMID: 36780674; PMCID: PMC10036605.

Altorki NK, Yip R, Hanaoka T, Bauer T, Aye R, Kohman L, Sheppard B, Thurer R, Andaz S, Smith M, Mayfield W, Grannis F, Korst R, Pass H, Straznicka M, Flores R, Henschke CI; I-ELCAP Investigators. Sublobar resection is equivalent to lobectomy for clinical stage 1A lung cancer in solid nodules. J Thorac Cardiovasc Surg. 2014 Feb;147(2):754-62; Discussion 762-4. doi: 10.1016/j.jtcvs.2013.09.065. Epub 2013 Nov 23. PMID: 24280722.

Annema JT, van Meerbeeck JP, Rintoul RC, Dooms C, Deschepper E, Dekkers OM, De Leyn P, Braun J, Carroll NR, Praet M, de Ryck F, Vansteenkiste J, Vermassen F, Versteegh MI, Veseliç M, Nicholson AG, Rabe KF, Tournoy KG. Mediastinoscopy vs endosonography for mediastinal nodal staging of lung cancer: a randomized trial. JAMA. 2010 Nov 24;304(20):2245-52. doi: 10.1001/jama.2010.1705. PMID: 21098770.

Annema JT, van Meerbeeck JP, Rintoul RC, Dooms C, Deschepper E, Dekkers OM, De Leyn P, Braun J, Carroll NR, Praet M, de Ryck F, Vansteenkiste J, Vermassen F, Versteegh MI, Veseliç M, Nicholson AG, Rabe KF, Tournoy KG. Mediastinoscopy vs endosonography for mediastinal nodal staging of lung cancer: a randomized trial. JAMA. 2010 Nov 24;304(20):2245-52. doi: 10.1001/jama.2010.1705. PMID: 21098770.

Asamura H, Chansky K, Crowley J, Goldstraw P, Rusch VW, Vansteenkiste JF, Watanabe H, Wu YL, Zielinski M, Ball D, Rami-Porta R; International Association for the Study of Lung Cancer Staging and Prognostic Factors Committee, Advisory Board Members, and Participating Institutions. The International Association for the Study of Lung Cancer Lung Cancer Staging Project: Proposals for the Revision of the N Descriptors in the Forthcoming 8th Edition of the TNM Classification for Lung Cancer. J Thorac Oncol. 2015 Dec;10(12):1675-84. doi: 10.1097/JTO.0000000000000678. PMID: 26709477.

Asamura H, Suzuki K, Kondo H, Tsuchiya R. Where is the boundary between N1 and N2 stations in lung cancer? Ann Thorac Surg. 2000 Dec;70(6):1839-45; discussion 1845-6. doi: 10.1016/s0003-4975(00)01817-8. PMID: 11156081.

Bai W, Zhang J, Wang Y, Zhou M, Liu L, Wang G, Zhao K, Gao X, Li S. Comparative analysis of the long-term outcomes of segmentectomy and lobectomy for stage IA1 lung adenocarcinoma in patients with or without previous malignancy of other organs: a population-based study. Expert Rev Anticancer Ther. 2022 Feb;22(2):215-228. doi: 10.1080/14737140.2021.1988570. Epub 2021 Oct 21. PMID: 34596477.

Baig MZ, Razi SS, Muslim Z, Weber JF, Connery CP, Bhora FY. Lobectomy Demonstrates Superior Survival Than Segmentectomy for High-Grade Non-Small Cell Lung Cancer: The National Cancer Database Analysis. Am Surg. 2023 Jan;89(1):120-128. doi: 10.1177/00031348211011116. Epub 2021 Apr 20. PMID: 33876966.

Bakir M, Fraser S, Routledge T, Scarci M. Is surgery indicated in patients with stage IIIa lung cancer and mediastinal nodal involvement? Interact Cardiovasc Thorac Surg. 2011 Sep;13(3):303-10. doi: 10.1510/icvts.2011.267872. Epub 2011 Jun 17. PMID: 21685220.

Behera M, Steuer CE, Liu Y, Fernandez F, Fu C, Higgins KA, Gillespie TW, Pakkala S, Pillai RN, Force S, Belani CP, Khuri FR, Curran WJ, Ramalingam SS. Trimodality Therapy in the Treatment of Stage III N2-Positive Non-Small Cell Lung Cancer: A National Cancer Database Analysis. Oncologist. 2020 Jun;25(6):e964-e975. doi: 10.1634/theoncologist.2019-0661. Epub 2020 Jan 14. PMID: 31943520; PMCID: PMC7288644.

Bendixen M, Jørgensen OD, Kronborg C, Andersen C, Licht PB. Postoperative pain and quality of life after lobectomy via video-assisted thoracoscopic surgery or anterolateral thoracotomy for early stage lung cancer: a randomised controlled trial. Lancet Oncol. 2016 Jun;17(6):836-844. doi: 10.1016/S1470-2045(16)00173-X. Epub 2016 May 6. PMID: 27160473.

Bendzsak AM, Waddell TK, Urbach DR, Darling GE. Surgery and Surgical Consult Rates for Early Stage Lung Cancer in Ontario: A Population-Based Study. Ann Thorac Surg. 2017 Mar;103(3):906-910. doi: 10.1016/j.athoracsur.2016.09.025. Epub 2016 Dec 7. PMID: 27939011.

Benoit L, Anusca A, Ortega-Deballon P, Cheynel N, Bernard A, Favre JP. Analysis of risk factors for skip lymphatic metastasis and their prognostic value in operated N2 non-small-cell lung carcinoma. Eur J Surg Oncol. 2006 Jun;32(5):583-7. doi: 10.1016/j.ejso.2006.02.004. Epub 2006 Apr 18. PMID: 16621424.

Berry MF, Worni M, Pietrobon R, D'Amico TA, Akushevich I. Variability in the treatment of elderly patients with stage IIIA (N2) non- small-cell lung cancer. J Thorac Oncol. 2013 Jun;8(6):744-52. doi: 10.1097/JTO.0b013e31828916aa. PMID: 23571473; PMCID: PMC3656972.

Berthet JP, Boada M, Paradela M, Molins L, Matecki S, Marty-Ané CH, Gómez-Caro A. Pulmonary sleeve resection in locally advanced lung cancer using cryopreserved allograft for pulmonary artery replacement. J Thorac Cardiovasc Surg. 2013 Nov;146(5):1191-7. doi: 10.1016/j.jtcvs.2013.07.003. Epub 2013 Aug 15. PMID: 23953718.

Berzenji L, Beckers P, Van Schil PE. Surgery for stage IIIA-N2 non-small cell lung cancer: the jury is still out! J Thorac Dis. 2019 May;11(Suppl 9):S1153-S1156. doi: 10.21037/jtd.2019.04.36. PMID: 31245070; PMCID: PMC6560604.

Billmeier SE, Ayanian JZ, Zaslavsky AM, Nerenz DR, Jaklitsch MT, Rogers SO. Predictors and outcomes of limited resection for early- stage non-small cell lung cancer. J Natl Cancer Inst. 2011 Nov 2;103(21):1621-9. doi: 10.1093/jnci/djr387. Epub 2011 Sep 29. PMID: 21960708; PMCID: PMC3206042.

Bitenc M, Cufer T, Kern I, Miklavcic M, Petrovic S, Groznik V, Sadikov A. Real-life long-term outcomes of upfront surgery in patients with resectable stage I-IIIA non-small cell lung cancer. Radiol Oncol. 2022 Aug 14;56(3):346-354. doi: 10.2478/raon-2022-0030. PMID: 35962955; PMCID: PMC9400448.

Bollen EC, Theunissen PH, van Duin CJ, Drenth BM, van Noord JA, Blijham GH. Clinical significance of intranodal and extranodal growth in lymph node metastases of non-small cell lung cancer. Scand J Thorac Cardiovasc Surg. 1994;28(3-4):97-102. doi: 10.3109/14017439409099112. PMID: 7792562.

Bousema JE, Heineman DJ, Dijkgraaf MGW, Annema JT, van den Broek FJC. Adherence to the mediastinal staging guideline and unforeseen N2 disease in patients with resectable non-small cell lung cancer: Nationwide results from the Dutch Lung Cancer Audit - Surgery. Lung Cancer. 2020 Apr;142:51-58. doi: 10.1016/j.lungcan.2020.02.008. Epub 2020 Feb 15. PMID: 32088606.

Bousema JE, van Dorp M, Noyez VJJM, Dijkgraaf MGW, Annema JT, van den Broek FJC. Unforeseen N2 Disease after Negative Endosonography Findings with or without Confirmatory Mediastinoscopy in Resectable Non-Small Cell Lung Cancer: A Systematic Review and Meta-Analysis. J Thorac Oncol. 2019 Jun;14(6):979-992. doi: 10.1016/j.jtho.2019.02.032. Epub 2019 Mar 21. PMID: 30905829.

Brichkov I, Keller SM. Intraoperative staging and surgical management of stage IIIA/N2 non-small cell lung cancer. Thorac Surg Clin. 2008 Nov;18(4):381-91. doi: 10.1016/j.thorsurg.2008.08.003. PMID: 19086607.

Bryan DS, Donington JS. The Role of Surgery in Management of Locally Advanced Non-Small Cell Lung Cancer. Curr Treat Options Oncol. 2019 Mar 14;20(4):27. doi: 10.1007/s11864-019-0624-7. PMID: 30874964.

Bryant AK, Mundt RC, Sandhu AP, Urbanic JJ, Sharabi AB, Gupta S, Daly ME, Murphy JD. Stereotactic Body Radiation Therapy Versus Surgery for Early Lung Cancer Among US Veterans. Ann Thorac Surg. 2018 Feb;105(2):425-431. doi: 10.1016/j.athoracsur.2017.07.048. Epub 2017 Nov 30. PMID: 29198624.

Bugalho A, Ferreira D, Barata R, Rodrigues C, Dias SS, Medeiros F, Carreiro L. Punção aspirativa transbrônquica guiada por ecoendoscopia brônquica no diagnóstico e estadiamento de cancro do pulmão em 179 doentes [Endobronchial ultrasound-guided transbronchial needle aspiration for lung cancer diagnosis and staging in 179 patients]. Rev Port Pneumol. 2013 Sep-Oct;19(5):192-9. Portuguese. doi: 10.1016/j.rppneu.2012.10.006. Epub 2013 Jul 12. PMID: 23850376.

Bugge AS, Lund MB, Valberg M, Brustugun OT, Solberg S, Kongerud J. Cause-specific death after surgical resection for early-stage non- small-cell lung cancer. Eur J Cardiothorac Surg. 2018 Jan 1;53(1):221-227. doi: 10.1093/ejcts/ezx274. PMID: 28950311.

Carr LL, Finigan JH, Kern JA. Evaluation and treatment of patients with non-small cell lung cancer. Med Clin North Am. 2011 Nov;95(6):1041-54. doi: 10.1016/j.mcna.2011.08.001. PMID: 22032426.

Casali C, Stefani A, Natali P, Rossi G, Morandi U. Prognostic factors in surgically resected N2 non-small cell lung cancer: the importance of patterns of mediastinal lymph nodes metastases. Eur J Cardiothorac Surg. 2005 Jul;28(1):33-8. doi: 10.1016/j.ejcts.2005.03.016. PMID: 15953734.

Casiraghi M, Galetta D, Borri A, Tessitore A, Romano R, Diotti C, Brambilla D, Maisonneuve P, Spaggiari L. Ten Years' Experience in Robotic-Assisted Thoracic Surgery for Early Stage Lung Cancer. Thorac Cardiovasc Surg. 2019 Oct;67(7):564-572. doi: 10.1055/s-0038- 1639575. Epub 2018 Apr 1. PMID: 29605962.

Caupena C, Costa R, Pérez-Ochoa F, Call S, Jaen À, Rami-Porta R, Obiols C, Esteban L, Albero-González R, Luizaga LA, Serra M, Belda J, Tarroch X, Sanz-Santos J. Nodal size ranking as a predictor of mediastinal involvement in clinical early-stage non-small cell lung cancer. Medicine (Baltimore). 2019 Dec;98(50):e18208. doi: 10.1097/MD.0000000000018208. PMID: 31852077; PMCID: PMC6922489.

Cerfolio RJ, Bryant AS, Minnich DJ. Complete thoracic mediastinal lymphadenectomy leads to a higher rate of pathologically proven N2 disease in patients with non-small cell lung cancer. Ann Thorac Surg. 2012 Sep;94(3):902-6. doi: 10.1016/j.athoracsur.2012.05.034. Epub 2012 Jul 7. PMID: 22776083.

Cetinkaya E, Turna A, Yildiz P, Dodurgali R, Bedirhan MA, Gürses A, Yilmaz V. Comparison of clinical and surgical-pathologic staging of the patients with non-small cell lung carcinoma. Eur J Cardiothorac Surg. 2002 Dec;22(6):1000-5. doi: 10.1016/s1010-7940(02)00581-x. PMID: 12467827.

Chambers A, Routledge T, Pilling J, Scarci M. In elderly patients with lung cancer is resection justified in terms of morbidity, mortality and residual quality of life? Interact Cardiovasc Thorac Surg. 2010 Jun;10(6):1015-21. doi: 10.1510/icvts.2010.233189. Epub 2010 Mar 30. PMID: 20354037.

Chen K, Yang F, Jiang G, Li J, Wang J. Development and validation of a clinical prediction model for N2 lymph node metastasis in non- small cell lung cancer. Ann Thorac Surg. 2013 Nov;96(5):1761-8. doi: 10.1016/j.athoracsur.2013.06.038. Epub 2013 Aug 30. PMID: 23998401.

Chida M. Surgery for T4N0-1 non-small cell lung cancer: from research to clinical practice. Ann Thorac Cardiovasc Surg. 2012;18(3):186- 7. doi: 10.5761/atcs.ed.12.01916. PMID: 22790987.

Cho S, Song IH, Yang HC, Kim K, Jheon S. Predictive factors for node metastasis in patients with clinical stage I non-small cell lung cancer. Ann Thorac Surg. 2013 Jul;96(1):239-45. doi: 10.1016/j.athoracsur.2013.03.050. Epub 2013 May 11. PMID: 23673071.

Chong CF, Khoo KL, Lim TK, Chang AY, Lim HL, Lee CN, Wong PS. Comparison of clinical with pathological nodal staging from systematic mediastinal lymph node dissection in early resectable non-small cell lung cancer. Singapore Med J. 2007 Jul;48(7):620-4. PMID: 17609822.

Citak N, Buyukkale S, Kok A, Celikten A, Metin M, Sayar A, Gurses A. Does video-assisted mediastinoscopy offer lower false-negative rates for subcarinal lymph nodes compared with standard cervical mediastinoscopy? Thorac Cardiovasc Surg. 2014 Oct;62(7):624-30. doi: 10.1055/s-0033-1358656. Epub 2013 Dec 2. PMID: 24297632.

Dai C, Shen J, Ren Y, Zhong S, Zheng H, He J, Xie D, Fei K, Liang W, Jiang G, Yang P, Petersen RH, Ng CS, Liu CC, Rocco G, Brunelli A, Shen Y, Chen C, He J. Choice of Surgical Procedure for Patients With Non-Small-Cell Lung Cancer ≤ 1 cm or > 1 to 2 cm Among Lobectomy, Segmentectomy, and Wedge Resection: A Population-Based Study. J Clin Oncol. 2016 Sep 10;34(26):3175-82. doi: 10.1200/JCO.2015.64.6729. Epub 2016 Jul 5. PMID: 27382092.

Dai Y, Long H, Lin P, Fu JH, Zhang LJ, Zhu ZH, Zhang X, Rong TH, Su XD. [Impact of the number of resected and involved lymph nodes on the outcome in patients with stage II non-small cell lung cancer]. Zhonghua Zhong Liu Za Zhi. 2010 Jun;32(6):436-40. Chinese. PMID: 20819485.

Darling GE. Lymph node assessment in early stage non-small cell lung cancer lymph node dissection or sampling? Gen Thorac Cardiovasc Surg. 2020 Jul;68(7):716-724. doi: 10.1007/s11748-020-01345-y. Epub 2020 Apr 7. PMID: 32266699.

D'Cunha J, Herndon JE 2nd, Herzan DL, Patterson GA, Kohman LJ, Harpole DH, Kernstine KH, Kern JA, Green MR, Maddaus MA, Kratzke RA; Cancer and Leukemia Group B. Poor correspondence between clinical and pathologic staging in stage 1 non-small cell lung cancer: results from CALGB 9761, a prospective trial. Lung Cancer. 2005 May;48(2):241-6. doi: 10.1016/j.lungcan.2004.11.006. Epub 2005 Jan 4. PMID: 15829324.

De Leyn P, Dooms C, Kuzdzal J, Lardinois D, Passlick B, Rami-Porta R, Turna A, Van Schil P, Venuta F, Waller D, Weder W, Zielinski M. Revised ESTS guidelines for preoperative mediastinal lymph node staging for non-small-cell lung cancer. Eur J Cardiothorac Surg. 2014 May;45(5):787-98. doi: 10.1093/ejcts/ezu028. Epub 2014 Feb 26. PMID: 24578407.

Decaluwé H, Moons J, Fieuws S, De Wever W, Deroose C, Stanzi A, Depypere L, Nackaerts K, Coolen J, Lambrecht M, Verbeken E, De Ruysscher D, Vansteenkiste J, Van Raemdonck D, De Leyn P, Dooms C; Leuven Lung Cancer Group. Is central lung tumour location really predictive for occult mediastinal nodal disease in (suspected) non-small-cell lung cancer staged cN0 on 18F-fluorodeoxyglucose positron emission tomography-computed tomography? Eur J Cardiothorac Surg. 2018 Jul 1;54(1):134-140. doi: 10.1093/ejcts/ezy018. PMID: 29447330.

Decaluwé H, Petersen RH, Brunelli A, Pompili C, Seguin-Givelet A, Gust L, Aigner C, Falcoz PE, Rinieri P, Augustin F, Sokolow Y, Verhagen A, Depypere L, Papagiannopoulos K, Gossot D, D'Journo XB, Guerrera F, Baste JM, Schmid T, Stanzi A, Van Raemdonck D, Bardet J, Thomas PA, Massard G, Fieuws S, Moons J, Dooms C, De Leyn P, Hansen HJ; MITIG-ESTS. Multicentric evaluation of the impact of central tumour location when comparing rates of N1 upstaging in patients undergoing video-assisted and open surgery for clinical Stage I non-small-cell lung cancer†. Eur J Cardiothorac Surg. 2018 Feb 1;53(2):359-365. doi: 10.1093/ejcts/ezx338. PMID: 29029062.

Decaluwé H, Stanzi A, Dooms C, Fieuws S, Coosemans W, Depypere L, Deroose CM, Dewever W, Nafteux P, Peeters S, Van Veer H, Verbeken E, Van Raemdonck D, Moons J, De Leyn P; Leuven Lung Cancer Group. Central tumour location should be considered when comparing N1 upstaging between thoracoscopic and open surgery for clinical stage I non-small-cell lung cancer. Eur J Cardiothorac Surg. 2016 Jul;50(1):110-7. doi: 10.1093/ejcts/ezv489. Epub 2016 Jan 27. PMID: 26819286.

Dell'Amore A, Monteverde M, Martucci N, Sanna S, Caroli G, Dolci G, Dell'Amore D, Rocco G. Lobar and sub-lobar lung resection in octogenarians with early stage non-small cell lung cancer: factors affecting surgical outcomes and long-term results. Gen Thorac Cardiovasc Surg. 2015 Apr;63(4):222-30. doi: 10.1007/s11748-014-0493-8. Epub 2014 Nov 18. PMID: 25403998.

Dell'Amore A, Monteverde M, Martucci N, Sanna S, Caroli G, Stella F, Dell'Amore D, Rocco G. Early and long-term results of pulmonary resection for non-small-cell lung cancer in patients over 75 years of age: a multi-institutional study. Interact Cardiovasc Thorac Surg. 2013 Mar;16(3):250-6. doi: 10.1093/icvts/ivs473. Epub 2012 Nov 23. PMID: 23178392; PMCID: PMC3568799.

Deng HY, Zhou J, Wang RL, Jiang R, Qiu XM, Zhu DX, Tang XJ, Zhou Q. Age-different extent of resection for clinical IA non-small cell lung cancer: analysis of nodal metastasis. Sci Rep. 2020 Jun 12;10(1):9587. doi: 10.1038/s41598-020-66509-5. PMID: 32533050; PMCID: PMC7293256.

Deng HY, Zhou J, Wang RL, Jiang R, Qiu XM, Zhu DX, Tang XJ, Zhou Q. Surgical Choice for Clinical Stage IA Non-Small Cell Lung Cancer: View From Regional Lymph Node Metastasis. Ann Thorac Surg. 2020 Apr;109(4):1079-1085. doi: 10.1016/j.athoracsur.2019.10.056. Epub 2019 Dec 14. PMID: 31846634.

Denlinger CE, Fernandez F, Meyers BF, Pratt W, Zoole JB, Patterson GA, Krupnick AS, Kreisel D, Crabtree T. Lymph node evaluation in video-assisted thoracoscopic lobectomy versus lobectomy by thoracotomy. Ann Thorac Surg. 2010 Jun;89(6):1730-5; discussion 1736. doi: 10.1016/j.athoracsur.2010.02.094. PMID: 20494019.

Detterbeck F. What to do with "Surprise" N2?: intraoperative management of patients with non-small cell lung cancer. J Thorac Oncol. 2008 Mar;3(3):289-302. doi: 10.1097/JTO.0b013e3181630ebd. PMID: 18317073.

Dolan DP, White A, Mazzola E, Lee DN, Gill R, Kucukak S, Bueno R, Jaklitsch MT, Mentzer SJ, Swanson SJ. Outcomes of superior segmentectomy versus lower lobectomy for superior segment Stage I non-small-cell lung cancer are equivalent: An analysis of 196 patients at a single, high volume institution. J Surg Oncol. 2021 Feb;123(2):570-578. doi: 10.1002/jso.26304. Epub 2020 Dec 1. PMID: 33259656.

Donahoe LL, de Valence M, Atenafu EG, Hanna WC, Waddell TK, Pierre AF, Yasufuku K, de Perrot M, Cypel M, Keshavjee S, Darling GE. High Risk for Thoracotomy but not Thoracoscopic Lobectomy. Ann Thorac Surg. 2017 Jun;103(6):1730-1735. doi: 10.1016/j.athoracsur.2016.11.076. Epub 2017 Mar 3. PMID: 28262299.

Dong S, Roberts SA, Chen S, Zhong X, Yang S, Qu X, Xu S. Survival after lobectomy versus sub-lobar resection in elderly with stage I NSCLC: a meta-analysis. BMC Surg. 2019 Apr 15;19(1):38. doi: 10.1186/s12893-019-0500-1. PMID: 30987622; PMCID: PMC6466711.

Dooms C, Tournoy KG, Schuurbiers O, Decaluwe H, De Ryck F, Verhagen A, Beelen R, van der Heijden E, De Leyn P. Endosonography for mediastinal nodal staging of clinical N1 non-small cell lung cancer: a prospective multicenter study. Chest. 2015 Jan;147(1):209-215. doi: 10.1378/chest.14-0534. PMID: 25211526.

Dziedzic DA, Cackowski MM, Zbytniewski M, Gryszko GM, Woźnica K, Orłowski TM; Polish Lung Cancer Study Group (PLCSG). The influence of the number of lymph nodes removed on the accuracy of a newly proposed N descriptor classification in patients with surgically- treated lung cancer. Surg Oncol. 2021 Jun;37:101514. doi: 10.1016/j.suronc.2020.12.008. Epub 2021 Jan 6. PMID: 33429325.

Evison M, Edwards T, Balata H, Tempowski A, Teng B, Bishop P, Fontaine E, Krysiak P, Rammohan K, Shah R, Crosbie P, Booton R. Prevalence of nodal metastases in lymph node stations 8 & 9 in a large UK lung cancer surgical centre without routine pre-operative EUS nodal staging. Lung Cancer. 2018 Jan;115:127-130. doi: 10.1016/j.lungcan.2017.11.023. Epub 2017 Nov 23. PMID: 29290254.

Fan XL, Liu YX, Tian H. Video-assisted thoracoscopic surgery for treatment of early- stage non-small cell lung cancer. Asian Pac J Cancer Prev. 2013;14(5):2871-7. doi: 10.7314/apjcp.2013.14.5.2871. PMID: 23803046.

Farrow NE, An SJ, Speicher PJ, Harpole DH Jr, D'Amico TA, Klapper JA, Hartwig MG, Tong BC. Disparities in guideline-concordant treatment for node-positive, non-small cell lung cancer following surgery. J Thorac Cardiovasc Surg. 2020 Jul;160(1):261-271.e1. doi: 10.1016/j.jtcvs.2019.10.102. Epub 2019 Nov 13. PMID: 31924363; PMCID: PMC7319034.

Ferguson, M., 2003. Optimal management when unsuspected N2 nodal disease is identified during thoracotomy for lung cancer: cost- effectiveness analysis. The Journal of Thoracic and Cardiovascular Surgery, 126(6), pp.1935-1942.

Fontaine E, McShane J, Carr M, Shackcloth M, Mediratta N, Page R, Poullis M. Should we operate on microscopic N2 non-small cell lung cancer? Interact Cardiovasc Thorac Surg. 2011 Jun;12(6):956-61; discussion 961. doi: 10.1510/icvts.2010.255323. Epub 2011 Feb 17. PMID: 21330443.

Francis S, Orton A, Stoddard G, Tao R, Hitchcock YJ, Akerley W, Kokeny KE. Sequencing of Postoperative Radiotherapy and Chemotherapy for Locally Advanced or Incompletely Resected Non-Small-Cell Lung Cancer. J Clin Oncol. 2018 Feb 1;36(4):333-341. doi: 10.1200/JCO.2017.74.4771. Epub 2017 Dec 13. PMID: 29236592.

Fukui T, Kato K, Okasaka T, Kawaguchi K, Fukumoto K, Nakamura S, Hakiri S, Ozeki N, Yokoi K. Predictors for hilar/intrapulmonary lymph node metastasis in discrete type of clinical N1 non-small cell lung cancer. Gen Thorac Cardiovasc Surg. 2017 Nov;65(11):640-645. doi: 10.1007/s11748-017-0827-4. Epub 2017 Sep 2. PMID: 28866794.

Fukui T, Mori S, Yokoi K, Mitsudomi T. Significance of the number of positive lymph nodes in resected non-small cell lung cancer. J Thorac Oncol. 2006 Feb;1(2):120-5. PMID: 17409840.

Gaer JA, Goldstraw P. Intraoperative assessment of nodal staging at thoracotomy for carcinoma of the bronchus. Eur J Cardiothorac Surg. 1990;4(4):207-10. doi: 10.1016/1010-7940(90)90006-l. PMID: 2185799.

Gao HJ, Jiang ZH, Gong L, Ma K, Ren P, Yu ZT, Wei YC. Video-Assisted Vs Thoracotomy Sleeve Lobectomy for Lung Cancer: A Propensity Matched Analysis. Ann Thorac Surg. 2019 Oct;108(4):1072-1079. doi: 10.1016/j.athoracsur.2019.04.037. Epub 2019 Jun 1. PMID: 31163131.

Gao SJ, Kim AW, Puchalski JT, Bramley K, Detterbeck FC, Boffa DJ, Decker RH. Indications for invasive mediastinal staging in patients with early non-small cell lung cancer staged with PET-CT. Lung Cancer. 2017 Jul;109:36-41. doi: 10.1016/j.lungcan.2017.04.018. Epub 2017 Apr 25. PMID: 28577947.

Geller AD, Zheng H, Mathisen DJ, Wright CD, Lanuti M. Relative incremental costs of complications of lobectomy for stage I non-small cell lung cancer. J Thorac Cardiovasc Surg. 2018 Apr;155(4):1804-1811. doi: 10.1016/j.jtcvs.2017.11.025. Epub 2017 Nov 20. PMID: 29254638.

Ghaly G, Kamel M, Nasar A, Paul S, Lee PC, Port JL, Stiles BM, Altorki NK. Video-Assisted Thoracoscopic Surgery Is a Safe and Effective Alternative to Thoracotomy for Anatomical Segmentectomy in Patients With Clinical Stage I Non-Small Cell Lung Cancer. Ann Thorac Surg. 2016 Feb;101(2):465-72; discussion 472. doi: 10.1016/j.athoracsur.2015.06.112. Epub 2015 Sep 26. PMID: 26391692.

Gómez-Caro A, Garcia S, Reguart N, Arguis P, Sanchez M, Gimferrer JM, Marrades R, Lomeña F. Incidence of occult mediastinal node involvement in cN0 non-small-cell lung cancer patients after negative uptake of positron emission tomography/computer tomography scan. Eur J Cardiothorac Surg. 2010 May;37(5):1168-74. doi: 10.1016/j.ejcts.2009.12.013. Epub 2010 Jan 29. PMID: 20116273.

Gorai A, Sakao Y, Kuroda H, Uehara H, Mun M, Ishikawa Y, Nakagawa K, Masuda M, Okumura S. The clinicopathological features associated with skip N2 metastases in patients with clinical stage IA non-small-cell lung cancer. Eur J Cardiothorac Surg. 2015 Apr;47(4):653-8. doi: 10.1093/ejcts/ezu244. Epub 2014 Jun 23. PMID: 24957260.

Graham AN, Chan KJ, Pastorino U, Goldstraw P. Systematic nodal dissection in the intrathoracic staging of patients with non-small cell lung cancer. J Thorac Cardiovasc Surg. 1999 Feb;117(2):246-51. doi: 10.1016/S0022-5223(99)70419-8. PMID: 9918964.

Guerrera F, Renaud S, Tabbó F, Voegeli' AC, Filosso PL, Legrain M, Boita M, Schaeffer M, Beau-Faller M, Ruffini E, Falcoz PE, Inghirami G, Oliaro A, Massard G. Epidermal growth factor receptor mutations are linked to skip N2 lymph node metastasis in resected non-small-cell lung cancer adenocarcinomas. Eur J Cardiothorac Surg. 2017 Apr 1;51(4):680-688. doi: 10.1093/ejcts/ezw362. PMID: 28329143.

Gunluoglu MZ, Demir A, Turna A, Sansar D, Melek H, Dincer SI, Gurses A. Extent of lung resection in non-small lung cancer with interlobar lymph node involvement. Ann Thorac Cardiovasc Surg. 2011;17(3):229-35. doi: 10.5761/atcs.oa.09.01530. PMID: 21697782.

Gürses A, Turna A, Bedirhan MA, Ozalp T, Kocatürk C, Demir A, Ozcan M, Urer N. The value of mediastinoscopy in preoperative evaluation of mediastinal involvement in non-small-cell lung cancer patients with clinical NO disease. Thorac Cardiovasc Surg. 2002 Jun;50(3):174-7. doi: 10.1055/s-2002-32416. PMID: 12077692.

Hanagiri T, Takenaka M, Oka S, Shigematsu Y, Nagata Y, Shimokawa H, Uramoto H, Tanaka F. Clinical significance in the number of involved lymph nodes in patients that underwent surgery for pathological stage III-N2 non-small cell lung cancer. J Cardiothorac Surg. 2011 Oct 25;6:144. doi: 10.1186/1749-8090-6-144. PMID: 22027105; PMCID: PMC3212931.

Handa Y, Tsutani Y, Mimae T, Miyata Y, Okada M. Surgical Procedure Selection for Stage I Lung Cancer: Complex Segmentectomy versus Wedge Resection. Clin Lung Cancer. 2021 Mar;22(2):e224-e233. doi: 10.1016/j.cllc.2020.10.021. Epub 2020 Nov 17. PMID: 33334701.

Hao X, Li W, Li W, Gu M, Wang Z, Nakahashi K, Antonoff MB, Adachi H, Zhou S, Xu S. Re-evaluating the need for mediastinal lymph node dissection and exploring lncRNAs as biomarkers of N2 metastasis in T1 lung adenocarcinoma. Transl Lung Cancer Res. 2022 Jun;11(6):1079-1088. doi: 10.21037/tlcr-22-207. PMID: 35832449; PMCID: PMC9271436.

Heineman DJ, Beck N, Wouters MW, van Brakel TJ, Daniels JM, Schreurs WH, Dickhoff C. The dutch national clinical audit for lung cancer: A tool to improve clinical practice? An analysis of unforeseen ipsilateral mediastinal lymph node involvement in the Dutch Lung Surgery Audit (DLSA). Eur J Surg Oncol. 2018 Jun;44(6):830-834. doi: 10.1016/j.ejso.2017.12.002. Epub 2018 Jan 9. PMID: 29396329.

Heineman DJ, Ten Berge MG, Daniels JM, Versteegh MI, Marang-van de Mheen PJ, Wouters MW, Schreurs WH. Clinical Staging of Stage I Non-Small Cell Lung Cancer in the Netherlands-Need for Improvement in an Era With Expanding Nonsurgical Treatment Options: Data From the Dutch Lung Surgery Audit. Ann Thorac Surg. 2016 Nov;102(5):1615-1621. doi: 10.1016/j.athoracsur.2016.07.054. Epub 2016 Sep 21. PMID: 27665481.

Heineman DJ, Ten Berge MG, Daniels JM, Versteegh MI, Marang-van de Mheen PJ, Wouters MW, Schreurs WH. The Quality of Staging Non-Small Cell Lung Cancer in the Netherlands: Data From the Dutch Lung Surgery Audit. Ann Thorac Surg. 2016 Nov;102(5):1622-1629. doi: 10.1016/j.athoracsur.2016.06.071. Epub 2016 Sep 21. PMID: 27665479.

Hennon MW, DeGraaff LH, Groman A, Demmy TL, Yendamuri S. The association of nodal upstaging with surgical approach and its impact on long-term survival after resection of non-small-cell lung cancer. Eur J Cardiothorac Surg. 2020 May 1;57(5):888-895. doi: 10.1093/ejcts/ezz320. PMID: 31764992; PMCID: PMC7179045.

Higgins KA, Chino JP, Ready N, D'Amico TA, Berry MF, Sporn T, Boyd J, Kelsey CR. Lymphovascular invasion in non-small-cell lung cancer: implications for staging and adjuvant therapy. J Thorac Oncol. 2012 Jul;7(7):1141-7. doi: 10.1097/JTO.0b013e3182519a42. PMID: 22617241.

Higuchi M, Yaginuma H, Yonechi A, Kanno R, Ohishi A, Suzuki H, Gotoh M. Long-term outcomes after video-assisted thoracic surgery (VATS) lobectomy versus lobectomy via open thoracotomy for clinical stage IA non-small cell lung cancer. J Cardiothorac Surg. 2014 May 17;9:88. doi: 10.1186/1749-8090-9-88. PMID: 24886655; PMCID: PMC4058716.

Hoang JK, Patz E Jr, Giroux D, Goldstraw P. Frequency and prognostic significance of preoperatively detected enlarged regional lymph nodes in patients with pathological stage I non-small cell lung cancer following resection. J Thorac Oncol. 2007 Dec;2(12):1103-6. doi: 10.1097/JTO.0b013e31815c04b4. PMID: 18090582.

Hoeijmakers F, Heineman DJ, Beck N, Klamer J, Tollenaar RAEM, Wouters MWJM, Schreurs WH. Mediastinoscopy for Staging of Non- Small Cell Lung Cancer: Surgical Performance in The Netherlands. Ann Thorac Surg. 2019 Apr;107(4):1024-1031. doi: 10.1016/j.athoracsur.2018.11.030. Epub 2018 Dec 14. PMID: 30557538.

Honguero Martínez AF, García Jiménez MD, García Vicente A, Genovés Crespo M, Rodríguez Ortega CR, Lázaro Sahuquillo M, Soriano Castrejón ÁM, León Atance P. Is the prognosis of occult N2 disease similar to that of positive positron emission tomography-computed tomography (PET/CT) scan single-station N2 disease in patients with non-small cell lung cancer treated by surgical resection? Rev Esp Med Nucl Imagen Mol. 2017 Nov-Dec;36(6):350-355. English, Spanish. doi: 10.1016/j.remn.2017.03.011. Epub 2017 May 26. PMID: 28552624.

Honguero Martínez AF, García Jiménez MD, García Vicente A, López-Torres Hidalgo J, Colon MJ, van Gómez López O, Soriano Castrejón ÁM, León Atance P. Ratio between maximum standardized uptake value of N1 lymph nodes and tumor predicts N2 disease in patients with non-small cell lung cancer in 18F-FDG PET-CT scan. Rev Esp Med Nucl Imagen Mol. 2016 May-Jun;35(3):159-64. English, Spanish. doi: 10.1016/j.remn.2015.08.004. Epub 2015 Oct 26. PMID: 26514322.

Huang CS, Hsu PK, Chen CK, Yeh YC, Hsu HS, Shih CC, Huang BS. Surgeons' preference sublobar resection for stage I NSCLC less than 3 cm. Thorac Cancer. 2020 Apr;11(4):907-917. doi: 10.1111/1759-7714.13336. Epub 2020 Feb 9. Erratum in: Thorac Cancer. 2021 Oct;12(19):2630. PMID: 32037690; PMCID: PMC7113050.

Huang KY, Chen HJ, Lin CH, Wang BY, Cheng CY, Lin SH. Comparison of recurrence risk between patients with clinically node-positive and -negative stage I non-small cell lung cancer following surgery: A propensity score matching analysis. Thorac Cancer. 2022 Jul;13(13):1933-1939. doi: 10.1111/1759-7714.14462. Epub 2022 May 17. PMID: 35581675; PMCID: PMC9250836.

Huang W, Deng HY, Ren ZZ, Xu K, Wang YF, Tang X, Zhu DX, Zhou Q. LobE-Specific lymph node diSsectiON for clinical early-stage non-small cell lung cancer: protocol for a randomised controlled trial (the LESSON trial). BMJ Open. 2022 Aug 29;12(8):e056043. doi: 10.1136/bmjopen-2021-056043. PMID: 36038163; PMCID: PMC9438114.

Hughes MJ, Chowdhry MF, Woolley SM, Walker WS. In patients undergoing lung resection for non-small cell lung cancer, is lymph node dissection or sampling superior? Interact Cardiovasc Thorac Surg. 2011 Sep;13(3):311-5. doi: 10.1510/icvts.2011.268979. Epub 2011 May 22. PMID: 21606053.

Husain ZA, Kim AW, Yu JB, Decker RH, Corso CD. Defining the High-Risk Population for Mortality After Resection of Early Stage NSCLC. Clin Lung Cancer. 2015 Nov;16(6):e183-7. doi: 10.1016/j.cllc.2015.04.007. Epub 2015 Apr 24. PMID: 25979646.

Hüyük M, Fiocco M, Postmus PE, Cohen D, von der Thüsen JH. Systematic review and meta-analysis of the prognostic impact of lymph node micrometastasis and isolated tumour cells in patients with stage I-IIIA non-small cell lung cancer. Histopathology. 2023 Apr;82(5):650-663. doi: 10.1111/his.14831. Epub 2022 Nov 9. PMID: 36282087.

Hwang Y, Kang CH, Kim HS, Jeon JH, Park IK, Kim YT. Comparison of thoracoscopic segmentectomy and thoracoscopic lobectomy on the patients with non-small cell lung cancer: a propensity score matching study. Eur J Cardiothorac Surg. 2015 Aug;48(2):273-8. doi: 10.1093/ejcts/ezu422. Epub 2014 Nov 18. PMID: 25406426.

Ichinokawa H, Takamochi K, Fukui M, Hattori A, Matsunaga T, Suzuki K. Surgical results and prognosis of lung cancer in elderly Japanese patients aged over 85 years: comparison with patients aged 80-84 years. Gen Thorac Cardiovasc Surg. 2021 Jan;69(1):67-75. doi: 10.1007/s11748-020-01426-y. Epub 2020 Jul 5. PMID: 32627148.

Ijsseldijk MA, Shoni M, Siegert C, Seegers J, van Engelenburg AKC, Tsai TC, Lebenthal A, Ten Broek RPG. Oncological Outcomes of Lobar Resection, Segmentectomy, and Wedge Resection for T1a Non-Small-Cell Lung Carcinoma: A Systematic Review and Meta- Analysis. Semin Thorac Cardiovasc Surg. 2020 Autumn;32(3):582-590. doi: 10.1053/j.semtcvs.2019.08.004. Epub 2019 Aug 9. PMID: 31401180.

IJsseldijk MA, Ten Broek RPG, Wiering B, Hekma E, de Roos MAJ. Oncological outcomes of unsuspected pN2 in patients with non-small- cell lung cancer: a systematic review and meta-analysis. Interact Cardiovasc Thorac Surg. 2021 May 10;32(5):727-736. doi: 10.1093/icvts/ivaa334. PMID: 33517373.

Ikeda K, Nomori H, Mori T, Kobayashi H, Iwatani K, Yoshimoto K. Size of metastatic and nonmetastatic mediastinal lymph nodes in non- small cell lung cancer. J Thorac Oncol. 2006 Nov;1(9):949-52. PMID: 17409977.

Im Y, Park HY, Shin S, Shin SH, Lee H, Ahn JH, Sohn I, Cho JH, Kim HK, Zo JI, Shim YM, Lee HY, Kim J. Prevalence of and risk factors for pulmonary complications after curative resection in otherwise healthy elderly patients with early stage lung cancer. Respir Res. 2019 Jul 4;20(1):136. doi: 10.1186/s12931-019-1087-x. PMID: 31272446; PMCID: PMC6610954.

Iskender I, Kadioglu SZ, Cosgun T, Kapicibasi HO, Sagiroglu G, Kosar A, Kir A. False-positivity of mediastinal lymph nodes has negative effect on survival in potentially resectable non-small cell lung cancer. Eur J Cardiothorac Surg. 2012 Apr;41(4):874-9. doi: 10.1093/ejcts/ezr054. Epub 2011 Nov 16. PMID: 22423060.

Iskender I, Kapicibasi HO, Kadioglu SZ, Sevilgen G, Tezel C, Kosar' A, Atasalihi A, Kir A. Comparison of integrated positron emission tomography/computed tomography and mediastinoscopy in mediastinal staging of non-small cell lung cancer: analysis of 212 patients. Acta Chir Belg. 2012 May-Jun;112(3):219-25. PMID: 22808763.

Ito H, Nakayama H, Yamada K, Yokose T, Masuda M. Outcomes of lobectomy in 'active' octogenarians with clinical stage I non-small-cell lung cancer. Ann Thorac Cardiovasc Surg. 2015;21(1):24-30. doi: 10.5761/atcs.oa.13-00353. Epub 2014 Apr 18. PMID: 24747546; PMCID: PMC4989983.

Izbicki JR, Passlick B, Karg O, Bloechle C, Pantel K, Knoefel WT, Thetter O. Impact of radical systematic mediastinal lymphadenectomy on tumor staging in lung cancer. Ann Thorac Surg. 1995 Jan;59(1):209-14. doi: 10.1016/0003-4975(94)00717-L. PMID: 7818326.

Jeon HW, Kim YD, Kim KS, Sung SW, Park HJ, Park JK. Sublobar resection versus lobectomy in solid-type, clinical stage IA, non-small cell lung cancer. World J Surg Oncol. 2014 Jul 16;12:215. doi: 10.1186/1477-7819-12-215. PMID: 25027055; PMCID: PMC4115487.

Jeremić B, Casas F, Dubinsky P, Gomez-Caamano A, Čihorić N, Videtic G. Surgery for Stage IIIA Non-Small-cell Lung Cancer: Lack of Predictive and Prognostic Factors Identifying Any Subgroup of Patients Benefiting From It. Clin Lung Cancer. 2016 Mar;17(2):107-12. doi: 10.1016/j.cllc.2015.11.001. Epub 2015 Nov 11. PMID: 26683387.

Jiang L, Jiang S, Lin Y, Yang H, Xie Z, Lin Y, Long H. Nomogram to Predict Occult N2 Lymph Nodes Metastases in Patients With Squamous Nonsmall Cell Lung Cancer. Medicine (Baltimore). 2015 Nov;94(46):e2054. doi: 10.1097/MD.0000000000002054. PMID: 26579815; PMCID: PMC4652824.

Jordá Aragón C, Peñalver Cuesta JC, Mancheño Franch N, de Aguiar Quevedo K, Vera Sempere F, Padilla Alarcón J. Mortalidad en carcinoma pulmonar no microcítico resecado, con tamaño máximo de 3 cm y sin afectación ganglionar: análisis de riesgos competitivos [Mortality in early-stage, surgically resected non-small cell lung cancer less than 3 cm of size: Competing risk analysis]. Med Clin (Barc). 2015 Sep 7;145(5):185-91. Spanish. doi: 10.1016/j.medcli.2014.07.032. Epub 2014 Nov 26. PMID: 25433784.

Kamigaichi A, Tsutani Y, Fujiwara M, Mimae T, Miyata Y, Okada M. Postoperative Recurrence and Survival After Segmentectomy for Clinical Stage 0 or IA Lung Cancer. Clin Lung Cancer. 2019 Sep;20(5):397-403.e1. doi: 10.1016/j.cllc.2019.06.004. Epub 2019 Jun 13. PMID: 31281050.

Kamigaichi A, Tsutani Y, Mimae T, Miyata Y, Shimada Y, Ito H, Nakayama H, Ikeda N, Okada M. Prediction of Unexpected N2 Disease Associated With Clinical T1-2N0-1M0 Non-Small-Cell Lung Cancer. Clin Lung Cancer. 2021 Mar;22(2):120-126.e3. doi: 10.1016/j.cllc.2020.12.010. Epub 2020 Dec 27. PMID: 33485802.

Kang CH, Ra YJ, Kim YT, Jheon SH, Sung SW, Kim JH. The impact of multiple metastatic nodal stations on survival in patients with resectable N1 and N2 nonsmall-cell lung cancer. Ann Thorac Surg. 2008 Oct;86(4):1092-7. doi: 10.1016/j.athoracsur.2008.06.056. PMID: 18805138.

Kang DY, Lee S. Lymphatic vessel invasion and lymph node metastasis in patients with clinical stage I non-small cell lung cancer. Thorac Cardiovasc Surg. 2014 Sep;62(6):521-4. doi: 10.1055/s-0034-1381744. Epub 2014 Jun 6. Erratum in: Thorac Cardiovasc Surg. 2014 Aug;62(5):e1. PMID: 24905019.

Kawachi R, Tsukada H, Nakazato Y, Takei H, Koshi-ishi Y, Goya T. Morbidity in video-assisted thoracoscopic lobectomy for clinical stage I non-small cell lung cancer: is VATS lobectomy really safe?

Thorac Cardiovasc Surg. 2009 Apr;57(3):156-9. doi: 10.1055/s-2008- 1039267. Epub 2009 Mar 27. PMID: 19330753.

Kilic A, Schuchert MJ, Pettiford BL, Pennathur A, Landreneau JR, Landreneau JP, Luketich JD, Landreneau RJ. Anatomic segmentectomy for stage I non-small cell lung cancer in the elderly. Ann Thorac Surg. 2009 Jun;87(6):1662-6; discussion 1667-8. doi: 10.1016/j.athoracsur.2009.02.097. PMID: 19463574.

Kim AW, Detterbeck FC, Boffa DJ, Decker RH, Soulos PR, Cramer LD, Gross CP. Characteristics associated with the use of nonanatomic resections among Medicare patients undergoing resections of early-stage lung cancer. Ann Thorac Surg. 2012 Sep;94(3):895-901. doi: 10.1016/j.athoracsur.2012.04.091. Epub 2012 Jul 25. PMID: 22835558; PMCID: PMC3501201.

Kim ES, Bosquée L. The importance of accurate lymph node staging in early and locally advanced non-small cell lung cancer: an update on available techniques. J Thorac Oncol. 2007 Jun;2 Suppl 2:S59-67. doi: 10.1097/01.JTO.0000269738.13586.fd. PMID: 17589301.

Kim MS, Lee HS, Lee JM, Zo JI, Lee GK, Nam BH. Prognostic value of single nodal zone metastasis in non-small-cell lung cancer. Eur J Cardiothorac Surg. 2010 Oct;38(4):491-7. doi: 10.1016/j.ejcts.2010.02.033. PMID: 20399672.

Kim, E. and Bosquée, L., 2007. The Importance of Accurate Lymph Node Staging in Early and Locally Advanced Non-small Cell Lung Cancer: An Update on Available Techniques. Journal of Thoracic Oncology, 2(6), pp.S59-S67.

Kono SA, Weyant M, Franklin W, Gaspar LE, Camidge DR. Surprised by stage III: unexpected N2 lymph node involvement found during surgery for early-stage NSCLC. Oncology (Williston Park). 2009 Apr 30;23(5):424-9. PMID: 19476276.

Kuroda H, Ichinose J, Masago K, Takahashi Y, Nakada T, Nakao M, Okumura S, Hashimoto K, Matsuura Y, Sakakura N, Matsushita H, Mun M. Permissible Outcomes of Lobe-Specific Lymph Node Dissection for Elevated Carcinoembryonic Antigen in Non-Small Cell Lung Cancer. Medicina (Kaunas). 2021 Dec 14;57(12):1365. doi: 10.3390/medicina57121365. PMID: 34946309; PMCID: PMC8709178.

Kuzdzał J, Zieliński M, Papla B, Szlubowski A, Hauer Ł, Nabiałek T, Sośnicki W, Pankowski J. Transcervical extended mediastinal lymphadenectomy--the new operative technique and early results in lung cancer staging. Eur J Cardiothorac Surg. 2005 Mar;27(3):384-90; discussion 390. doi: 10.1016/j.ejcts.2004.12.008. Epub 2005 Jan 13. PMID: 15740943.

Lagerwaard FJ, Verstegen NE, Haasbeek CJ, Slotman BJ, Paul MA, Smit EF, Senan S. Outcomes of stereotactic ablative radiotherapy in patients with potentially operable stage I non-small cell lung cancer. Int J Radiat Oncol Biol Phys. 2012 May 1;83(1):348-53. doi: 10.1016/j.ijrobp.2011.06.2003. Epub 2011 Nov 19. PMID: 22104360.

Lautamäki A, Gunn J, Sipilä J, Rautava P, Sihvo E, Kytö V. Women have a higher resection rate for lung cancer and improved survival after surgery. Interact Cardiovasc Thorac Surg. 2021 May 27;32(6):889-895. doi: 10.1093/icvts/ivab006. PMID: 33523210.

Lee BE, Redwine J, Foster C, Abella E, Lown T, Lau D, Follette D. Mediastinoscopy might not be necessary in patients with non-small cell lung cancer with mediastinal lymph nodes having a maximum standardized uptake value of less than 5.3. J Thorac Cardiovasc Surg. 2008 Mar;135(3):615-9. doi: 10.1016/j.jtcvs.2007.09.029. Epub 2008 Jan 11. PMID: 18329480.

Lee HS, Jang HJ. Thoracoscopic mediastinal lymph node dissection for lung cancer. Semin Thorac Cardiovasc Surg. 2012 Summer;24(2):131-41. doi: 10.1053/j.semtcvs.2012.02.004. PMID: 22920530.

Lee J, Hong YS, Cho J, Lee J, Lee G, Kang D, Yun J, Jeon YJ, Shin S, Cho JH, Choi YS, Kim J, Zo JI, Shim YM, Guallar E, Kim HK. Reclassifying the International Association for the Study of Lung Cancer Residual Tumor Classification According to the Extent of Nodal Dissection for NSCLC: One Size Does Not Fit All. J Thorac Oncol. 2022 Jul;17(7):890-899. doi: 10.1016/j.jtho.2022.03.015. Epub 2022 Apr 21. PMID: 35462086.

Lee J, Kim HK, Park BJ, Cho JH, Choi YS, Zo JI, Shim YM, Pyo H, Ahn YC, Ahn JS, Ahn MJ, Park K, Kim J. Recurrence dynamics after trimodality therapy (Neoadjuvant concurrent chemoradiotherapy and surgery) in patients with stage IIIA (N2) lung cancer. Lung Cancer. 2018 Jan;115:89-96. doi: 10.1016/j.lungcan.2017.11.020. Epub 2017 Nov 22. PMID: 29290268.

Lee K, Jeong YH, Ryu JS, Kim YI, Kim HR, Park SI. Surgical Outcomes of Non-Small Cell Lung Cancer in Single-Zone N2 in the Aortopulmonary Zone. Thorac Cardiovasc Surg. 2022 Apr;70(3):251-257. doi: 10.1055/s-0041-1727206. Epub 2021 Apr 21. PMID: 33882612.

Lee PC, Nasar A, Port JL, Paul S, Stiles B, Chiu YL, Andrews WG, Altorki NK. Long-term survival after lobectomy for non-small cell lung cancer by video-assisted thoracic surgery versus thoracotomy. Ann Thorac Surg. 2013 Sep;96(3):951-60; discussion 960-1. doi: 10.1016/j.athoracsur.2013.04.104. Epub 2013 Jul 16. PMID: 23866808.

Lee PC, Port JL, Korst RJ, Liss Y, Meherally DN, Altorki NK. Risk factors for occult mediastinal metastases in clinical stage I non-small cell lung cancer. Ann Thorac Surg. 2007 Jul;84(1):177-81. doi: 10.1016/j.athoracsur.2007.03.081. PMID: 17588407.

Li S, Wang H, Yang Z, Zhao L, Lv W, Du H, Che G, Liu L. Naples Prognostic Score as a novel prognostic prediction tool in video-assisted thoracoscopic surgery for early-stage lung cancer: a propensity score matching study. Surg Endosc. 2021 Jul;35(7):3679-3697. doi: 10.1007/s00464-020-07851-7. Epub 2020 Aug 3. PMID: 32748268.

Liberman M, Sampalis J, Duranceau A, Thiffault V, Hadjeres R, Ferraro P. Endosonographic mediastinal lymph node staging of lung cancer. Chest. 2014 Aug;146(2):389-397. doi: 10.1378/chest.13-2349. PMID: 24603902.

Lin JT, Yang XN, Zhong WZ, Liao RQ, Dong S, Nie Q, Weng SX, Fang XJ, Zheng JY, Wu YL. Association of maximum standardized uptake value with occult mediastinal lymph node metastases in cN0 non-small cell lung cancer. Eur J Cardiothorac Surg. 2016 Nov;50(5):914-919. doi: 10.1093/ejcts/ezw109. Epub 2016 Apr 24. PMID: 27113090.

Lioumpas, D., Tomos, P., Zaragkas, S., Fillipou, D., Tzatzadakis, N., Michos, T., Fili, N., Stamatatos, I., Balakera, C., Samiotis, I., Bouboulis, K. and Kleontas, A., 2020. Deeper into the mediastinum: Topographical distribution and histological patterns of occult pN2 disease in NSCLC patients. Lung cancer.

Little AG, Rusch VW, Bonner JA, Gaspar LE, Green MR, Webb WR, Stewart AK. Patterns of surgical care of lung cancer patients. Ann Thorac Surg. 2005 Dec;80(6):2051-6; discussion 2056. doi: 10.1016/j.athoracsur.2005.06.071. PMID: 16305843.

Liu C, Pu Q, Guo C, Xiao Z, Mei J, Ma L, Zhu Y, Liao H, Liu L. Non-grasping en bloc mediastinal lymph node dissection for video- assisted thoracoscopic lung cancer surgery. BMC Surg. 2015 Apr 8;15:38. doi: 10.1186/s12893-015-0025-1. PMID: 25884998; PMCID: PMC4392751.

Lou F, Huang J, Sima CS, Dycoco J, Rusch V, Bach PB. Patterns of recurrence and second primary lung cancer in early-stage lung cancer survivors followed with routine computed tomography surveillance. J Thorac Cardiovasc Surg. 2013 Jan;145(1):75-81; discussion 81-2. doi: 10.1016/j.jtcvs.2012.09.030. Epub 2012 Nov 3. PMID: 23127371.

Lozekoot PWJ, Daemen JHT, van den Broek RR, Maessen JG, Gronenschild MHM, Vissers YLJ, Hulsewé KWE, de Loos ER. Surgical mediastinal lymph node staging for non-small-cell lung carcinoma. Transl Lung Cancer Res. 2021 Aug;10(8):3645-3658. doi: 10.21037/tlcr- 21-364. PMID: 34584863; PMCID: PMC8435384.

Lucchi M, Viti A, Melfi F, Ambrogi M, Givigliano F, Dini P, Mussi A. IIIB-T4 non-small cell lung cancer: indications and results of surgical treatment. J Cardiovasc Surg (Torino). 2007 Jun;48(3):369-74. PMID: 17505443.

Lv X, Cao J, Dai X, Rusidanmu A. Survival rates after lobectomy versus sublobar resection for early-stage right middle lobe non-small cell lung cancer. Thorac Cancer. 2018 Aug;9(8):1026-1031. doi: 10.1111/1759-7714.12782. Epub 2018 Jun 21. PMID: 29927089; PMCID: PMC6068437.

M. Prognostic factors for lymph node negative stage I and IIA non-small cell lung cancer: multicenter experiences. Asian Pac J Cancer Prev. 2013;14(11):6287-92. doi: 10.7314/apjcp.2013.14.11.6287. PMID: 24377519.

Ma Q, Liu D, Guo Y, Shi B, Song Z, Tian Y. Surgical therapeutic strategy for non-small cell lung cancer with mediastinal lymph node metastasis (N2). Zhongguo Fei Ai Za Zhi. 2010 Apr;13(4):342-8. doi: 10.3779/j.issn.1009-3419.2010.04.14. PMID: 20677562; PMCID: PMC6000432.

Ma Z, Dong A, Fan J, Cheng H. Does sleeve lobectomy concomitant with or without pulmonary artery reconstruction (double sleeve) have favorable results for non-small cell lung cancer compared with pneumonectomy? A meta-analysis. Eur J Cardiothorac Surg. 2007 Jul;32(1):20-8. doi: 10.1016/j.ejcts.2007.03.018. Epub 2007 Apr 17. PMID: 17442581.

Macia I, Ramos R, Moya J, et al. Survival of patients with non-small cell lung cancer according to lymph node disease: single pN1 vs multiple pN1 vs single unsuspected pN2. Ann Surg Oncol. 2013;20(7):2413-2418. doi:10.1245/s10434-012-2865-6

Maeda R, Yoshida J, Ishii G, Hishida T, Nishimura M, Nagai K. Risk factors for tumor recurrence in patients with early-stage (stage I and II) non-small cell lung cancer: patient selection criteria for adjuvant chemotherapy according to the seventh edition TNM classification. Chest. 2011 Dec;140(6):1494-1502. doi: 10.1378/chest.10-3279. Epub 2011 May 26. PMID: 21622548.

Mao F, Pan Y, Li Z, Cai M, Shen-Tu Y. [High risk indication of postoperative chemotherapy in early stage non-small cell lung cancer]. Zhongguo Fei Ai Za Zhi. 2014 May;17(5):411-6. Chinese. doi: 10.3779/j.issn.1009-3419.2014.05.09. PMID: 24854559; PMCID: PMC6000450.

Martin CM, Puello-Guerrero A, Mas-Lopez LA, Campos-Gómez S, Orlando-Orlandi FJ, Tejado Gallegos LF, Huggenberger R. Real-world KINDLE-Latin America subset data on treatment patterns and clinical outcomes in patients with stage III non-small-cell lung cancer. Cancer Med. 2023 Jan;12(2):1247-1259. doi: 10.1002/cam4.4990. Epub 2022 Jul 4. PMID: 35789068; PMCID: PMC9883579.

Martini N, Flehinger BJ, Zaman MB, Beattie EJ Jr. Results of resection in non-oat cell carcinoma of the lung with mediastinal lymph node metastases. Ann Surg. 1983 Sep;198(3):386-97. doi: 10.1097/00000658-198309000-00015. PMID: 6615059; PMCID: PMC1353314.

Matsuura N, Go T, Fujiwara A, Nakano T, Nakashima N, Tarumi S, Chang SS, Yokomise H. Lymphatic invasion is a cause of local recurrence after wedge resection of primary lung cancer. Gen Thorac Cardiovasc Surg. 2019 Oct;67(10):861-866. doi: 10.1007/s11748-019- 01095-6. Epub 2019 Feb 28. PMID: 30820912.

Mayne NR, Darling AJ, Raman V, Balderson S, Berry MF, Harpole DH Jr, D'Amico TA, Yang CJ. Perioperative Outcomes and 5-year Survival After Open versus Thoracoscopic Sleeve Resection for Lung Cancer. Semin Thorac Cardiovasc Surg. 2021 Summer;33(2):522- 530. doi: 10.1053/j.semtcvs.2020.08.013. Epub 2020 Aug 25. PMID: 32858216.

McElnay PJ, Choong A, Jordan E, Song F, Lim E. Outcome of surgery versus radiotherapy after induction treatment in patients with N2 disease: systematic review and meta-analysis of randomised trials. Thorax. 2015 Aug;70(8):764-8. doi: 10.1136/thoraxjnl-2014-206292. Epub 2015 May 12. Erratum in: Thorax. 2019 Aug;74(8):824. PMID: 25967753.

Merritt RE, Abdel-Rasoul M, Fitzgerald M, D'Souza DM, Kneuertz PJ. Nomograms for Predicting Overall and Recurrence-free Survival From Pathologic Stage IA and IB Lung Cancer After Lobectomy. Clin Lung Cancer. 2021 Jul;22(4):e574-e583. doi: 10.1016/j.cllc.2020.10.009. Epub 2020 Oct 22. PMID: 33234491.

Mimae T, Miyata Y, Tsutani Y, Imai K, Ito H, Nakayama H, Ikeda N, Okada M. Wedge resection as an alternative treatment for octogenarian and older patients with early-stage non-small-cell lung cancer. Jpn J Clin Oncol. 2020 Sep 5;50(9):1051-1057. doi: 10.1093/jjco/hyaa085. PMID: 32577731.

Mitzman B, Varghese TK Jr, Kuchta K, Krantz SB. National guideline concordance and outcomes for pathologic N2 disease in non-small cell lung cancer. J Thorac Dis. 2022 May;14(5):1360-1373. doi: 10.21037/jtd-21-1845. PMID: 35693597; PMCID: PMC9186219.

Miyazaki T, Yamazaki T, Nakamura D, Sato S, Yamasaki N, Tsuchiya T, Matsumoto K, Kamohara R, Hatachi G, Nagayasu T. Surgery or stereotactic body radiotherapy for elderly stage I lung cancer? A propensity score matching analysis. Surg Today. 2017 Dec;47(12):1476- 1483. doi: 10.1007/s00595-017-1536-4. Epub 2017 Apr 26. PMID: 28447170.

Miyoshi S, Shien K, Toyooka S, Miyoshi K, Yamamoto H, Sugimoto S, Soh J, Hayama M, Yamane M, Oto T. Validity of using lobe- specific regional lymph node stations to assist navigation during lymph node dissection in early stage non-small cell lung cancer patients. Surg Today. 2014 Nov;44(11):2028-36. doi: 10.1007/s00595-013-0772-5. Epub 2013 Oct 31. PMID: 24170276.

Monteiro AS, Araújo SRC, Araujo LH, Souza MC. Impact of microvascular invasion on 5-year overall survival of resected non-small cell lung cancer. J Bras Pneumol. 2022 Jul 8;48(3):e20210283. doi: 10.36416/1806-3756/e20210283. PMID: 35830051; PMCID: PMC9262425.

Murakawa T, Ichinose J, Hino H, Kitano K, Konoeda C, Nakajima J. Long-term outcomes of open and video-assisted thoracoscopic lung lobectomy for the treatment of early stage non-small cell lung cancer are similar: a propensity-matched study. World J Surg. 2015 May;39(5):1084-91. doi: 10.1007/s00268-014-2918-z. PMID: 25561187.

Na II, Park JY, Kim KM, Cheon GJ, Choe DH, Koh JS, Baek HJ, Lee JC. Significance of smoking history and FDG uptake for pathological N2 staging in clinical N2-negative non-small-cell lung cancer. Ann Oncol. 2011 Sep;22(9):2068-2072. doi: 10.1093/annonc/mdq693. Epub 2011 Jan 21. PMID: 21257671.

Nakagiri T, Sawabata N, Funaki S, Inoue M, Kadota Y, Shintani Y, Okumura M. Validation of pN2 sub-classifications in patients with pathological stage IIIA N2 non-small cell lung cancer. Interact Cardiovasc Thorac Surg. 2011 May;12(5):733-8. doi: 10.1510/icvts.2010.249896. Epub 2011 Feb 5. PMID: 21297135.

Nakajima T, Kimura H, Suzuki M, Wada H, Ando S, Iida T, Sekine Y, Fujisawa T. A prospective phase II study of a new treatment protocol for NSCLC combined with preoperative mediastinoscopy and chemotherapy. Thorac Cardiovasc Surg. 2007 Aug;55(5):317-21. doi: 10.1055/s-2006-955961. PMID: 17629863.

Naruke T, Goya T, Tsuchiya R, Suemasu K. The importance of surgery to non-small cell carcinoma of lung with mediastinal lymph node metastasis. Ann Thorac Surg. 1988 Dec;46(6):603-10. doi: 10.1016/s0003-4975(10)64717-0. PMID: 2848463.

Nicoli CD, Sprague BL, Anker CJ, Lester-Coll NH. Association of Rurality With Survival and Guidelines-Concordant Management in Early-stage Non-Small Cell Lung Cancer. Am J Clin Oncol. 2019 Jul;42(7):607-614. doi: 10.1097/COC.0000000000000549. PMID: 31232724.

Nonaka M, Kadokura M, Yamamoto S, Kataoka D, Kunimura T, Kushima M, Horichi N, Takaba T. Tumor dimension and prognosis in surgically treated lung cancer: for intentional limited resection. Am J Clin Oncol. 2003 Oct;26(5):499-503. doi: 10.1097/01.coc.0000037739.92442.52. PMID: 14528079.

Oda R, Okuda K, Osaga S, Watanabe T, Sakane T, Tatematsu T, Yokota K, Haneda H, Nakanishi R. Long-term outcomes of video-assisted thoracoscopic surgery lobectomy vs. thoracotomy lobectomy for stage IA non-small cell lung cancer. Surg Today. 2019 May;49(5):369-377. doi: 10.1007/s00595-018-1746-4. Epub 2018 Dec 3. PMID: 30511319.

Ohkubo T, Sugiura H, Itoh K, Ohno K, Morikawa T, Okushiba S, Kondoh S, Katoh H. [Surgical analysis for N 2 factor in non-small cell lung cancer]. Kyobu Geka. 2001 Jan;54(1):80-5. Japanese. PMID: 11197915.

Ohta Y, Shimizu Y, Minato H, Matsumoto I, Oda M, Watanabe G. Results of initial operations in non-small cell lung cancer patients with single-level N2 disease. Ann Thorac Surg. 2006 Feb;81(2):427-33. doi: 10.1016/j.athoracsur.2005.08.018. PMID: 16427826.

Ohtsuka T, Nomori H, Horio H, Naruke T, Suemasu K. Is major pulmonary resection by video-assisted thoracic surgery an adequate procedure in clinical stage I lung cancer? Chest. 2004 May;125(5):1742-6. doi: 10.1378/chest.125.5.1742. PMID: 15136385.

Okada M, Yamagishi H, Satake S, Matsuoka H, Miyamoto Y, Yoshimura M, Tsubota N. Survival related to lymph node involvement in lung cancer after sleeve lobectomy compared with pneumonectomy. J Thorac Cardiovasc Surg. 2000 Apr;119(4 Pt 1):814-9. doi: 10.1016/S0022-5223(00)70018-3. PMID: 10733774.

Okamoto T, Tagawa T, Morodomi Y, Shimamatsu S, Kitahara H, Maehara Y. Underlying Problems in Surgical Treatment of cT1-2N1 Non- Small Cell Lung Cancer. Thorac Cardiovasc Surg. 2017 Mar;65(2):130-135. doi: 10.1055/s-0035-1558648. Epub 2015 Aug 10. PMID: 26258472.

Okumura M, Goto M, Ideguchi K, Tamura M, Sasaki H, Tanaka H, Matsumura A, Iuchi K. Factors associated with outcome of segmentectomy for non-small cell lung cancer: long-term follow-up study at a single institution in Japan. Lung Cancer. 2007 Nov;58(2):231-7. doi: 10.1016/j.lungcan.2007.06.014. Epub 2007 Jul 30. PMID: 17673328.

Onkologie. 2013;36(9):492-6. doi: 10.1159/000354631. Epub 2013 Aug 19. PMID: 24051925.

Orlowski TM, Szczesny TJ. Surgical treatment of stage III non-small cell lung cancer. Lung Cancer. 2001 Dec;34 Suppl 2:S137-43. doi: 10.1016/s0169-5002(01)00358-0. PMID: 11720755.

Osarogiagbon RU, Allen JW, Farooq A, Berry A, O'Brien T. Pathologic lymph node staging practice and stage-predicted survival after resection of lung cancer. Ann Thorac Surg. 2011 May;91(5):1486-92. doi: 10.1016/j.athoracsur.2010.11.065. Erratum in: Ann Thorac Surg. 2011 Oct;92(4):1556. PMID: 21524460.

Osarogiagbon RU, Lee YS, Faris NR, Ray MA, Ojeabulu PO, Smeltzer MP. Invasive mediastinal staging for resected non-small cell lung cancer in a population-based cohort. J Thorac Cardiovasc Surg. 2019 Oct;158(4):1220-1229.e2. doi: 10.1016/j.jtcvs.2019.04.068. Epub 2019 May 2. PMID: 31147169; PMCID: PMC6754300.

Osarogiagbon RU, Ramirez RA, Wang CG, Miller LE, Smeltzer MM, Sareen S, Javed AY, Robbins SG, Khandekar A, Wolf BA, Gibson J, Spencer D, Robbins ET. Dual intervention to improve pathologic staging of resectable lung cancer. Ann Thorac Surg. 2013 Dec;96(6):1975- 81. doi: 10.1016/j.athoracsur.2013.07.009. Epub 2013 Sep 23. PMID: 24067333.

Ose N, Takeuchi Y, Sakamaki Y, Kadota Y, Urasaki K, Tsuji H, Kawahara K, Noguchi M, Shintani Y. Detection of lymph node metastasis in non-small cell lung cancer using the new system of one-step nucleic acid amplification assay. PLoS One. 2022 Mar 21;17(3):e0265603. doi: 10.1371/journal.pone.0265603. PMID: 35312731; PMCID: PMC8936453.

Oven Ustaalioglu BB, Unal OU, Turan N, Bilici A, Kaya S, Eren T, Ulas A, Inal A, Berk V, Demirci U, Alici S, Bal O, Benekli M, Gumus

Palade E, Passlick B, Osei-Agyemang T, Günter J, Wiesemann S. Video-assisted vs open mediastinal lymphadenectomy for Stage I non- small-cell lung cancer: results of a prospective randomized trial. Eur J Cardiothorac Surg. 2013 Aug;44(2):244-9; discussion 249. doi: 10.1093/ejcts/ezs668. Epub 2013 Jan 7. PMID: 23295451.

Park BJ, Melfi F, Mussi A, Maisonneuve P, Spaggiari L, Da Silva RK, Veronesi G. Robotic lobectomy for non-small cell lung cancer (NSCLC): long-term oncologic results. J Thorac Cardiovasc Surg. 2012 Feb;143(2):383-9. doi: 10.1016/j.jtcvs.2011.10.055. Epub 2011 Nov 20. PMID: 22104677.

Park SY, Byun GE, Lee CY, Lee JG, Kim DJ, Paik HC, Chung KY. Clinical implications of uncertain resection in scenarios of metastasis of the highest or most distant mediastinal lymph node station following surgical treatment of non-small-cell lung cancer. Lung Cancer. 2019 Dec;138:1-5. doi: 10.1016/j.lungcan.2019.09.018. Epub 2019 Sep 25. PMID: 31593893.

Peng L, Shang QW, Deng HY, Liu ZK, Li W, Wang Y. Lobe-specific lymph node dissection in early-stage non-small-cell lung cancer: An overview. Asian J Surg. 2023 Feb;46(2):683-687. doi: 10.1016/j.asjsur.2022.07.042. Epub 2022 Jul 30. PMID: 35918226.

Petrosyan F, Daw H, Haddad A, Spiro T, Sood R. Gene expression profiling for early-stage NSCLC. Am J Clin Oncol. 2015 Feb;38(1):103- 7. doi: 10.1097/COC.0b013e31828d95d8. PMID: 23608827.

Pfannschmidt J, Kollmeier J. Ergebnisse der N1- und N2-Chirurgie beim nichtkleinzelligen Lungenkarzinom [Results of N1 and N2 surgery in non-small cell lung cancer]. Chirurg. 2019 Dec;90(12):974-981. German. doi: 10.1007/s00104-019-01029-1. PMID: 31501934.

Poncelet AJ, Cornet J, Coulon C, Collard P, Noirhomme P, Weynand B; groupe d'oncologie thoracique des Cliniques Saint-Luc. Intra- tumoral vascular or perineural invasion as prognostic factors for long-term survival in early stage non-small cell lung carcinoma. Eur J Cardiothorac Surg. 2008 May;33(5):799-804. doi: 10.1016/j.ejcts.2008.01.060. PMID: 18374590.

Pricopi C, Rivera C, Abdennadher M, Arame A, Foucault C, Dujon A, Le Pimpec Barthes F, Riquet M. Place des résections limitées et leurs facteurs pronostiques dans le cancer bronchique non à petites cellules [Place of limited resections and prognostic factors in non-small lung cancer]. Rev Pneumol Clin. 2015 Aug;71(4):207-16. French. doi: 10.1016/j.pneumo.2014.09.005. Epub 2015 Mar 18. PMID: 25794877.

Qiao GB, Zeng WS, Peng LJ, Jiang RC, Pang DZ, Peng XF, Wu YL. [Surgical treatment for bronchioloalveolar carcinoma with ipsilateral intrapulmonary metastatic nodules]. Zhonghua Zhong Liu Za Zhi. 2009 Aug;31(8):634-7. Chinese. PMID: 20021955.

Ray MA, Faris NR, Smeltzer MP, Fehnel C, Houston-Harris C, Levy P, Wiggins L, Sachdev V, Robbins T, Spencer D, Osarogiagbon RU. Effectiveness of Implemented Interventions on Pathologic Nodal Staging of Non-Small Cell Lung Cancer. Ann Thorac Surg. 2018 Jul;106(1):228-234. doi: 10.1016/j.athoracsur.2018.02.021. Epub 2018 Mar 11. PMID: 29534956; PMCID: PMC6019187.

Razi SS, Kodia K, Alnajar A, Block MI, Tarrazzi F, Nguyen D, Villamizar N. Lobectomy Versus Stereotactic Body Radiotherapy in Healthy Octogenarians With Stage I Lung Cancer. Ann Thorac Surg. 2021 May;111(5):1659-1665. doi: 10.1016/j.athoracsur.2020.06.097. Epub 2020 Sep 3. PMID: 32891656.

Riquet M, Assouad J, Bagan P, Foucault C, Le Pimpec Barthes F, Dujon A, Danel C. Skip mediastinal lymph node metastasis and lung cancer: a particular N2 subgroup with a better prognosis. Ann Thorac Surg. 2005 Jan;79(1):225-33. doi: 10.1016/j.athoracsur.2004.06.081. PMID: 15620948.

Riquet M, Bagan P, Le Pimpec Barthes F, Banu E, Scotte F, Foucault C, Dujon A, Danel C. Completely resected non-small cell lung cancer: reconsidering prognostic value and significance of N2 metastases. Ann Thorac Surg. 2007 Dec;84(6):1818-24. doi: 10.1016/j.athoracsur.2007.07.015. PMID: 18036891.

Riquet M, Legras A, Mordant P, Rivera C, Arame A, Gibault L, Foucault C, Dujon A, Le Pimpec Barthes F. Number of mediastinal lymph nodes in non-small cell lung cancer: a Gaussian curve, not a prognostic factor. Ann Thorac Surg. 2014 Jul;98(1):224-31. doi: 10.1016/j.athoracsur.2014.03.023. Epub 2014 May 10. PMID: 24820386.

Riquet M, Manac'h D, Le Pimpec Barthes F, Dujon A, Debrosse D, Debesse B. Prognostic value of T and N in non small cell lung cancer three centimeters or less in diameter. Eur J Cardiothorac Surg. 1997 Mar;11(3):440-3; discussion 443-4. doi: 10.1016/s1010- 7940(96)01019-6. PMID: 9105805.

Riquet M, Manac'h D, Saab M, Le Pimpec-Barthes F, Dujon A, Debesse B. Factors determining survival in resected N2 lung cancer. Eur J Cardiothorac Surg. 1995;9(6):300-4. doi: 10.1016/s1010-7940(05)80186-1. PMID: 7546801.

Rodrigus P. The impact of surgical adjuvant thoracic radiation for different stages of non-small cell lung cancer: the experience from a single institution. Lung Cancer. 1999 Jan;23(1):11-7. doi: 10.1016/s0169-5002(98)00099-3. PMID: 10100142.

Rogasch JMM, Frost N, Bluemel S, Michaels L, Penzkofer T, von Laffert M, Temmesfeld-Wollbrück B, Neudecker J, Rückert JC, Ochsenreither S, Böhmer D, Amthauer H, Furth C. FDG-PET/CT for

pretherapeutic lymph node staging in non-small cell lung cancer: A tailored approach to the ESTS/ESMO guideline workflow. Lung Cancer. 2021 Jul;157:66-74. doi: 10.1016/j.lungcan.2021.05.003. Epub 2021 May 7. PMID: 33994197.

Rud AK, Boye K, Fodstad Ø, Juell S, Jørgensen LH, Solberg S, Helland Å, Brustugun OT, Mælandsmo GM. Detection of disseminated tumor cells in lymph nodes from patients with early stage non-small cell lung cancer. Diagn Pathol. 2016 Jun 17;11(1):50. doi: 10.1186/s13000-016-0504-4. PMID: 27316334; PMCID: PMC4912762.

Rueth NM, Parsons HM, Habermann EB, Groth SS, Virnig BA, Tuttle TM, Andrade RS, Maddaus MA, D'Cunha J. Surgical treatment of lung cancer: predicting postoperative morbidity in the elderly population. J Thorac Cardiovasc Surg. 2012 Jun;143(6):1314-23. doi: 10.1016/j.jtcvs.2011.09.072. Epub 2012 Feb 15. PMID: 22341420.

Ruffini E, Filosso PL, Bruna MC, Coni F, Cristofori RC, Mossetti C, Solidoro P, Oliaro A. Recommended changes for T and N descriptors proposed by the International Association for the Study of Lung Cancer - Lung Cancer Staging Project: a validation study from a single- centre experience. Eur J Cardiothorac Surg. 2009 Dec;36(6):1037-44. doi: 10.1016/j.ejcts.2009.05.051. Epub 2009 Aug 18. PMID: 19692259.

Sakuraba M, Takahashi N, Oh S, Miyasaka Y, Inagaki T, Suzuki K. Long-term survival after complete mediastinal lymph node resection and lobectomy in patients with bulky N2 non-small cell lung cancer. Ann Thorac Cardiovasc Surg. 2011;17(2):124-9. doi: 10.5761/atcs.oa.09.01475. PMID: 21597408.

Samayoa AX, Pezzi TA, Pezzi CM, Greer Gay E, Asai M, Kulkarni N, Carp N, Chun SG, Putnam JB Jr. Rationale for a Minimum Number of Lymph Nodes Removed with Non-Small Cell Lung Cancer Resection: Correlating the Number of Nodes Removed with Survival in 98,970 Patients. Ann Surg Oncol. 2016 Dec;23(Suppl 5):1005-1011. doi: 10.1245/s10434-016-5509-4. Epub 2016 Aug 16. PMID: 27531307.

Samejima J, Nakao M, Matsuura Y, Uehara H, Mun M, ch K, Motoi N, Masuda M, Ishikawa Y, Okumura S. Prognostic impact of bulky swollen lymph nodes in cN1 non-small cell lung cancer patients. Jpn J Clin Oncol. 2015 Nov;45(11):1050-4. doi: 10.1093/jjco/hyv129. Epub 2015 Sep 9. PMID: 26355162.

Samson P, Crabtree T, Broderick S, Kreisel D, Krupnick AS, Patterson GA, Meyers B, Puri V. Quality Measures in Clinical Stage I Non- Small Cell Lung Cancer: Improved Performance Is Associated With Improved Survival. Ann Thorac Surg. 2017 Jan;103(1):303-311. doi: 10.1016/j.athoracsur.2016.07.003. Epub 2016 Sep 21. PMID: 27665480; PMCID: PMC5182109.

Sanborn RE, Lally BE. Adjuvant therapy for non-small cell lung cancer with mediastinal nodal involvement. Thorac Surg Clin. 2008 Nov;18(4):423-35. doi: 10.1016/j.thorsurg.2008.08.004. PMID: 19086611.

Sanz-Santos J, Martínez-Palau M, Jaen À, Rami-Porta R, Barreiro B, Call S, Obiols C, González JM, De Marcos JÁ, Ysamat M, Canales L, Serra M, Belda J. Geometrical Measurement of Central Tumor Location in cT1N0M0 NSCLC Predicts N1 but Not N2 Upstaging. Ann Thorac Surg. 2021 Apr;111(4):1190-1197. doi: 10.1016/j.athoracsur.2020.06.040. Epub 2020 Aug 24. PMID: 32853568.

Scheel PJ 3rd, Crabtree TD, Bell JM, Frederiksen C, Broderick SR, Krupnick AS, Kreisel D, Patterson GA, Meyers BF, Puri V. Does surgeon experience affect outcomes in pathologic stage I lung cancer? J

Thorac Cardiovasc Surg. 2015 Apr;149(4):998-1004.e1. doi: 10.1016/j.jtcvs.2014.12.032. Epub 2014 Dec 20. PMID: 25636526; PMCID: PMC4409482.

Schlachtenberger G, Doerr F, Menghesha H, Heldwein MB, Hagmeyer L, Michel M, Schaefer SC, Wahlers T, Hekmat K. Postoperative long-term survival of non-small cell lung cancer patients with skip-N2 metastases. Surg Oncol. 2021 Sep;38:101505. doi: 10.1016/j.suronc.2020.11.019. Epub 2020 Nov 29. PMID: 33901730.

Schuchert MJ, Abbas G, Pennathur A, Nason KS, Wilson DO, Luketich JD, Landreneau RJ. Sublobar resection for early-stage lung cancer. Semin Thorac Cardiovasc Surg. 2010 Spring;22(1):22-31. doi: 10.1053/j.semtcvs.2010.04.004. PMID: 20813313.

Schuchert MJ, Normolle DP, Awais O, Pennathur A, Wilson DO, Luketich JD, Landreneau RJ. Factors influencing recurrence following anatomic lung resection for clinical stage I non-small cell lung cancer. Lung Cancer. 2019 Feb;128:145-151. doi: 10.1016/j.lungcan.2018.12.026. Epub 2018 Dec 26. PMID: 30642447; PMCID: PMC7507791.

Scott WJ, Howington J, Feigenberg S, Movsas B, Pisters K; American College of Chest Physicians. Treatment of non-small cell lung cancer stage I and stage II: ACCP evidence-based clinical practice guidelines (2nd edition). Chest. 2007 Sep;132(3 Suppl):234S-242S. doi: 10.1378/chest.07-1378. PMID: 17873171.

Sebastian NT, Merritt RE, Abdel-Rasoul M, Wu T, Bazan JG, Xu-Welliver M, Haglund K, D'Souza D, Kneuertz PJ, Williams TM. Recurrence After Stereotactic Body Radiation Therapy Versus Lobectomy for Non-Small Cell Lung Cancer. Ann Thorac Surg. 2020 Sep;110(3):998-1005. doi: 10.1016/j.athoracsur.2020.03.073. Epub 2020 Apr 27. PMID: 32353436.

Seo YS, Kim HJ, Wu HG, Choi SM, Park S. Lobectomy versus stereotactic ablative radiotherapy for medically operable patients with stage IA non-small cell lung cancer: A virtual randomized phase III trial stratified by age. Thorac Cancer. 2019 Jun;10(6):1489-1499. doi: 10.1111/1759-7714.13103. Epub 2019 May 23. PMID: 31124275; PMCID: PMC6558457.

Seo YS, Kim HJ, Wu HG, Choi SM, Park S. Lobectomy versus stereotactic ablative radiotherapy for medically operable patients with stage IA non-small cell lung cancer: A virtual randomized phase III trial stratified by age. Thorac Cancer. 2019 Jun;10(6):1489-1499. doi: 10.1111/1759-7714.13103. Epub 2019 May 23. PMID: 31124275; PMCID: PMC6558457.

Seok Y, Jeong JY, Lee E. Extent of visceral pleural invasion and the prognosis of surgically resected node-negative non-small cell lung cancer. Thorac Cancer. 2017 May;8(3):197-202. doi: 10.1111/1759-7714.12424. Epub 2017 Feb 21. PMID: 28220643; PMCID: PMC5415476.

Sesti J, Donington JS. Managing lung cancer in high-risk patients: what to consider. Expert Rev Respir Med. 2014 Aug;8(4):443-52. doi: 10.1586/17476348.2014.918508. Epub 2014 Jun 6. PMID: 24905801.

Shagabayeva L, Fu B, Panda N, Potter AL, Auchincloss HG, Mansur A, Jeffrey Yang CF, Schumacher L. Open, Video- and Robot-Assisted Thoracoscopic Lobectomy for Stage II-IIIA Non-Small Cell Lung Cancer. Ann Thorac Surg. 2023 Jan;115(1):184-190. doi: 10.1016/j.athoracsur.2022.01.026. Epub 2022 Feb 8. PMID: 35149049.

Sharples LD, Jackson C, Wheaton E, Griffith G, Annema JT, Dooms C, Tournoy KG, Deschepper E, Hughes V, Magee L, Buxton M, Rintoul RC. Clinical effectiveness and cost-effectiveness of endobronchial and endoscopic ultrasound relative to surgical staging in potentially resectable lung

cancer: results from the ASTER randomised controlled trial. Health Technol Assess. 2012;16(18):1-75, iii-iv. doi: 10.3310/hta16180. PMID: 22472180.

Shien K, Toyooka S, Soh J, Okami J, Higashiyama M, Kadota Y, Maeda H, Hayama M, Chida M, Funaki S, Okumura M, Miyoshi S. Clinicopathological characteristics and lymph node metastasis pathway of non-small-cell lung cancer located in the left lingular division. Interact Cardiovasc Thorac Surg. 2015 Jun;20(6):791-6. doi: 10.1093/icvts/ivv062. Epub 2015 Mar 24. PMID: 25805399.

Shiono S, Abiko M, Sato T. Limited resection for clinical Stage IA non-small-cell lung cancers based on a standardized-uptake value index. Eur J Cardiothorac Surg. 2013 Jan;43(1):e7-e12. doi: 10.1093/ejcts/ezs573. Epub 2012 Nov 4. PMID: 23129358.

Shirvani SM, Jiang J, Chang JY, Welsh J, Likhacheva A, Buchholz TA, Swisher SG, Smith BD. Lobectomy, sublobar resection, and stereotactic ablative radiotherapy for early-stage non-small cell lung cancers in the elderly. JAMA Surg. 2014 Dec;149(12):1244-53. doi: 10.1001/jamasurg.2014.556. PMID: 25321323; PMCID: PMC4401470.

Shirvani SM, Jiang J, Chang JY, Welsh JW, Gomez DR, Swisher S, Buchholz TA, Smith BD. Comparative effectiveness of 5 treatment strategies for early-stage non-small cell lung cancer in the elderly. Int J Radiat Oncol Biol Phys. 2012 Dec 1;84(5):1060-70. doi: 10.1016/j.ijrobp.2012.07.2354. Epub 2012 Sep 11. PMID: 22975611; PMCID: PMC3776428.

Sigel K, Bonomi M, Packer S, Wisnivesky J. Effect of age on survival of clinical stage I non-small-cell lung cancer. Ann Surg Oncol. 2009 Jul;16(7):1912-7. doi: 10.1245/s10434-009-0475-8. Epub 2009 May 2. PMID: 19408051.

Sioris T, Järvenpää R, Kuukasjärvi P, Helin H, Saarelainen S, Tarkka M. Comparison of computed tomography and systematic lymph node dissection in determining TNM and stage in non-small cell lung cancer. Eur J Cardiothorac Surg. 2003 Mar;23(3):403-8. doi: 10.1016/s1010-7940(02)00806-0. PMID: 12614814.

Sirbu H, Schreiner W, Dalichau H, Busch T. Surgery for non-small cell carcinoma in geriatric patients: 15-year experience. Asian Cardiovasc Thorac Ann. 2005 Dec;13(4):330-6. doi: 10.1177/021849230501300408. PMID: 16304220.

Smeltzer MP, Faris NR, Ray MA, Osarogiagbon RU. Association of Pathologic Nodal Staging Quality With Survival Among Patients With Non-Small Cell Lung Cancer After Resection With Curative Intent. JAMA Oncol. 2018 Jan 1;4(1):80-87. doi: 10.1001/jamaoncol.2017.2993. PMID: 28973110; PMCID: PMC5833630.

Smith CB, Swanson SJ, Mhango G, Wisnivesky JP. Survival after segmentectomy and wedge resection in stage I non-small-cell lung cancer. J Thorac Oncol. 2013 Jan;8(1):73-8. doi: 10.1097/JTO.0b013e31827451c4. PMID: 23164939.

Smolle-Juettner FM, Maier A, Lindenmann J, Matzi V, Neuböck N. Resection in stage I/II non-small cell lung cancer. Front Radiat Ther Oncol. 2010;42:71-77. doi: 10.1159/000262462. Epub 2009 Nov 24. PMID: 19955793.

Smythe WR. Treatment of stage I and II non-small-cell lung cancer. Cancer Control. 2001 Jul-Aug;8(4):318-25. doi: 10.1177/107327480100800403. PMID: 11483885.

Sonobe M, Date H, Wada H, Okubo K, Hamakawa H, Teramukai S, Matsumura A, Nakagawa T, Sumitomo S, Miyamoto Y, Okumura N, Takeo S, Kawakami K, Aoki M, Kosaka S; The Japan-

Multinational Trial Organization. Prognostic factors after complete resection of pN2 non-small cell lung cancer. J Thorac Cardiovasc Surg. 2013 Oct;146(4):788-95. doi: 10.1016/j.jtcvs.2013.04.043. Epub 2013 Jun 27. PMID: 23810113.

Spaggiari L, Casiraghi M, Guarize J, Brambilla D, Petrella F, Maisonneuve P, De Marinis F. Outcome of Patients With pN2 "Potentially Resectable" Nonsmall Cell Lung Cancer Who Underwent Surgery After Induction Chemotherapy. Semin Thorac Cardiovasc Surg. 2016 Summer;28(2):593-602. doi: 10.1053/j.semtcvs.2015.12.001. Epub 2015 Dec 10. PMID: 28043483.

Stamatis G, Leschber G, Schwarz B, Brintrup DL, Flossdorf S, Passlick B, Hecker E, Kugler C, Eichhorn M, Krbek T, Eggeling S, Hatz R, Müller MR, Hillinger S, Aigner C, Jöckel KH. Survival outcomes in a prospective randomized multicenter Phase III trial comparing patients undergoing anatomical segmentectomy versus standard lobectomy for non-small cell lung cancer up to 2 cm. Lung Cancer. 2022 Oct;172:108-116. doi: 10.1016/j.lungcan.2022.08.013. Epub 2022 Aug 24. PMID: 36058174.

Stiles BM, Kamel MK, Nasar A, Harrison S, Nguyen AB, Lee P, Port JL, Altorki NK. The importance of lymph node dissection accompanying wedge resection for clinical stage IA lung cancer. Eur J Cardiothorac Surg. 2017 Mar 1;51(3):511-517. doi: 10.1093/ejcts/ezw343. PMID: 28007869.

Su X, Wang X, Long H, Fu J, Lin P, Zhang L, Wang S, Rong T. Mediastinal lymph node dissection affects survival in patients with stage I non-small cell lung cancer. Thorac Cardiovasc Surg. 2008 Jun;56(4):226-30. doi: 10.1055/s-2007-989494. PMID: 18481243.

Sugi K, Kaneda Y, Esato K. Video-assisted thoracoscopic lobectomy achieves a satisfactory long-term prognosis in patients with clinical stage IA lung cancer. World J Surg. 2000 Jan;24(1):27-30; discussion 30-1. doi: 10.1007/s002689910006. PMID: 10594199.

Sullivan KA, Farrokhyar F, Leontiadis GI, Patel YS, Churchill IF, Hylton DA, Xie F, Seely AJE, Spicer J, Kidane B, Turner SR, Yasufuku K, Hanna WC. Routine systematic sampling versus targeted sampling during endobronchial ultrasound: A randomized feasibility trial. J Thorac Cardiovasc Surg. 2022 Jul;164(1):254-261.e1. doi: 10.1016/j.jtcvs.2021.11.062. Epub 2021 Dec 4. PMID: 35031139.

Sun J, Wu S, Jin Z, Ren S, Cho WC, Zhu C, Shen J. Lymph node micrometastasis in non-small cell lung cancer. Biomed Pharmacother. 2022 May;149:112817. doi: 10.1016/j.biopha.2022.112817. Epub 2022 Mar 15. PMID: 35303567.

Suzuki K, Nagai K, Yoshida J, Nishimura M, Takahashi K, Nishiwaki Y. Clinical predictors of N2 disease in the setting of a negative computed tomographic scan in patients with lung cancer. J Thorac Cardiovasc Surg. 1999 Mar;117(3):593-8. doi: 10.1016/s0022- 5223(99)70340-5. PMID: 10047665.

Suzuki K, Nagai K, Yoshida J, Nishimura M, Takahashi K, Nishiwaki Y. The prognosis of surgically resected N2 non-small cell lung cancer: the importance of clinical N status. J Thorac Cardiovasc Surg. 1999 Jul;118(1):145-53. doi: 10.1016/S0022-5223(99)70153-4. PMID: 10384197.

Szlubowski A, Zieliński M, Soja J, Annema JT, Sośnicki W, Jakubiak M, Pankowski J, Cmiel A. A combined approach of endobronchial and endoscopic ultrasound-guided needle aspiration in the radiologically normal mediastinum in non-small-cell lung cancer staging--a prospective trial. Eur J Cardiothorac Surg. 2010 May;37(5):1175-9. doi: 10.1016/j.ejcts.2009.11.015. Epub 2009 Dec 22. PMID: 20022761.

Takamochi K, Nagai K, Suzuki K, Yoshida J, Ohde Y, Nishiwaki Y. Clinical predictors of N2 disease in non-small cell lung cancer. Chest. 2000 Jun;117(6):1577-82. doi: 10.1378/chest.117.6.1577. PMID: 10858386.

Takamochi K, Oh S, Suzuki K. Prognostic evaluation of nodal staging based on the new IASLC lymph node map for lung cancer. Thorac Cardiovasc Surg. 2010 Sep;58(6):345-9. doi: 10.1055/s-0030-1249944. Epub 2010 Sep 7. PMID: 20824587.

Tanaka F, Yanagihara K, Otake Y, Miyahara R, Kawano Y, Nakagawa T, Shoji T, Wada H. Surgery for non-small cell lung cancer: postoperative survival based on the revised tumor-node-metastasis classification and its time trend. Eur J Cardiothorac Surg. 2000 Aug;18(2):147-55. doi: 10.1016/s1010-7940(00)00490-5. PMID: 10925222.

Tanner NT, Gomez M, Rainwater C, Nietert PJ, Simon GR, Green MR, Silvestri GA. Physician preferences for management of patients with stage IIIA NSCLC: impact of bulk of nodal disease on therapy selection. J Thorac Oncol. 2012 Feb;7(2):365-9. doi: 10.1097/JTO.0b013e31823a385f. PMID: 22237260; PMCID: PMC3527069.

Taylor M, Evison M, Clayton B, Grant SW, Martin GP, Shah R, Krysiak P, Rammohan K, Fontaine E, Joshi V, Granato F. Adequacy of Mediastinal Lymph Node Sampling in Patients With Lung Cancer Undergoing Lung Resection. J Surg Res. 2022 Feb;270:271-278. doi: 10.1016/j.jss.2021.09.014. Epub 2021 Oct 27. PMID: 34715539.

Tezel C, Dogruyol T, Alpay L, Akyıl M, Evman S, Metin S, Baysungur V, Yalcinkaya I. Prognostic Importance of the Lymph Node Factor in Surgically Resected Non-Small Cell Lung Cancer. Thorac Cardiovasc Surg. 2020 Mar;68(2):183-189. doi: 10.1055/s-0038-1675345. Epub 2018 Nov 2. PMID: 30388719.

Thornblade LW, Mulligan MS, Odem-Davis K, Hwang B, Waworuntu RL, Wolff EM, Kessler L, Wood DE, Farjah F. Challenges in Predicting Recurrence After Resection of Node-Negative Non-Small Cell Lung Cancer. Ann Thorac Surg. 2018 Nov;106(5):1460-1467. doi: 10.1016/j.athoracsur.2018.06.022. Epub 2018 Jul 19. PMID: 30031845; PMCID: PMC6347463.

Tomaszek SC, Kim Y, Cassivi SD, Jensen MR, Shen KH, Nichols FC, Deschamps C, Wigle DA. Bronchial resection margin length and clinical outcome in non-small cell lung cancer. Eur J Cardiothorac Surg. 2011 Nov;40(5):1151-6. doi: 10.1016/j.ejcts.2011.02.042. Epub 2011 Mar 30. PMID: 21450488.

Tomita M, Matsuzaki Y, Shimizu T, Hara M, Ayabe T, Onitsuka T. Preoperative prognostic factors for pN2 non-small cell lung cancer. Ann Thorac Cardiovasc Surg. 2006 Feb;12(1):15-20. PMID: 16572069.

Tsai YM, Huang TW, Hsu HH, Cheng CY, Lin YC, Cheng YL, Chang H, Lee SC. Prognostic significance of the number of removed lymph nodes at lobectomy in patients with positron emission tomography-computed tomography-negative N2 non-small cell lung cancer.

Tsubochi H, Kanai Y, Nakano T, Koyama S, Sohara Y, Endo S. [Port-access lobectomy for lung cancer: experience in the single institute]. Kyobu Geka. 2009 Apr;62(4):267-70. Japanese. PMID: 19348208.

Tsukioka T, Nishiyama N, Iwata T, Izumi N, Mizuguchi S, Morita R, Inoue K, Suehiro S. Early recurrence of completely resected N2- positive non-small-cell lung cancer. Gen Thorac Cardiovasc Surg. 2007 Mar;55(3):113-8. doi: 10.1007/s11748-006-0082-6. PMID: 17447509.

Tsutani Y, Nakayama H, Ito H, Handa Y, Mimae T, Miyata Y, Okada M. Long-Term Outcomes After Sublobar Resection Versus Lobectomy in Patients With Clinical Stage IA Lung Adenocarcinoma Meeting the Node-Negative Criteria Defined by High-Resolution Computed Tomography and [18F]-Fluoro-2-Deoxy-d-Glucose Positron Emission Tomography. Clin Lung Cancer. 2021 May;22(3):e431-e437. doi: 10.1016/j.cllc.2020.06.013. Epub 2020 Jun 19. PMID: 32665166.

Turk F, Gursoy S, Yaldiz S, Yuncu G, Yazgan S, Basok O. Comparison of clinical and pathological tumor, node and metastasis staging of lung cancer: 15-year experience with 530 patients. Minerva Chir. 2011 Dec;66(6):509-16. PMID: 22233657.

Turna A, Melek H, Kara HV, Kılıç B, Erşen E, Kaynak K. Validity of the updated European Society of Thoracic Surgeons staging guideline in lung cancer patients. J Thorac Cardiovasc Surg. 2018 Feb;155(2):789-795. doi: 10.1016/j.jtcvs.2017.09.090. Epub 2017 Sep 27. PMID: 29110950.

Uehara H, Nakao M, Mun M, Nakagawa K, Nishio M, Ishikawa Y, Okumura S. Significant prognostic factors for completely resected pN2 non-small cell lung cancer without neoadjuvant therapy. Ann Thorac Cardiovasc Surg. 2015;21(4):345-53. doi: 10.5761/atcs.oa.14-00262. Epub 2015 Feb 16. PMID: 25740454; PMCID: PMC4904870.

van den Berg LL, Klinkenberg TJ, Groen HJM, Widder J. Patterns of Recurrence and Survival after Surgery or Stereotactic Radiotherapy for Early Stage NSCLC. J Thorac Oncol. 2015 May;10(5):826-831. doi: 10.1097/JTO.0000000000000483. PMID: 25629639.

van der Woude L, Wouters MWJM, Hartemink KJ, Heineman DJ, Verhagen AFTM. Completeness of lymph node dissection in patients undergoing minimally invasive- or open surgery for non-small cell lung cancer: A nationwide study. Eur J Surg Oncol. 2021 Jul;47(7):1784- 1790. doi: 10.1016/j.ejso.2020.11.008. Epub 2020 Nov 13. PMID: 33223414.

Van Schil PE. Optimal Treatment of Stage IIIA-N2 Non-Small Cell Lung Cancer: A Neverending Story? J Thorac Oncol. 2017 Sep;12(9):1338-1340. doi: 10.1016/j.jtho.2017.07.001. PMID: 28838709.

Van Schil PE. Stage IIIA-N2 non-small-cell lung cancer: from 'surprise' involvement to surgical nightmare. Eur J Cardiothorac Surg. 2016 Jun;49(6):1613-4. doi: 10.1093/ejcts/ezv457. Epub 2015 Dec 30. PMID: 26719400.

Vannucci F, Gonzalez-Rivas D. Is VATS lobectomy standard of care for operable non-small cell lung cancer? Lung Cancer. 2016 Oct;100:114-119. doi: 10.1016/j.lungcan.2016.08.004. Epub 2016 Aug 11. PMID: 27597290.

Vansteenkiste J, Crinò L, Dooms C, Douillard JY, Faivre-Finn C, Lim E, Rocco G, Senan S, Van Schil P, Veronesi G, Stahel R, Peters S, Felip E; Panel Members. 2nd ESMO Consensus Conference on Lung Cancer: early-stage non-small-cell lung cancer consensus on diagnosis, treatment and follow-up. Ann Oncol. 2014 Aug;25(8):1462-74. doi: 10.1093/annonc/mdu089. Epub 2014 Feb 20. PMID: 24562446.

Vansteenkiste JF, De Leyn PR, Deneffe GJ, Lerut TE, Demedts MG. Clinical prognostic factors in surgically treated stage IIIA-N2 non- small cell lung cancer: analysis of the literature. Lung Cancer. 1998 Jan;19(1):3-13. doi: 10.1016/s0169-5002(97)00072-x. PMID: 9493135.

Varlotto JM, Yao AN, DeCamp MM, Ramakrishna S, Recht A, Flickinger J, Andrei A, Reed MF, Toth JW, Fizgerald TJ, Higgins K, Zheng X, Shelkey J, Medford-Davis LN, Belani C, Kelsey CR. Nodal stage of surgically resected non-small cell lung cancer and its effect on recurrence patterns and overall survival. Int J Radiat Oncol Biol Phys. 2015 Mar 15;91(4):765-73. doi: 10.1016/j.ijrobp.2014.12.028. PMID: 25752390.

Veeramachaneni NK, Feins RH, Stephenson BJ, Edwards LJ, Fernandez FG. Management of stage IIIA non-small cell lung cancer by thoracic surgeons in North America. Ann Thorac Surg. 2012 Sep;94(3):922-6; discussion 926-8. doi: 10.1016/j.athoracsur.2012.04.087. Epub 2012 Jun 27. Erratum in: Ann Thorac Surg. 2013 Oct;96(4):1532. Erratum in: Ann Thorac Surg. 2013 Oct;96(4):1532. PMID: 22742842.

Veluswamy RR, Whittaker Brown SA, Mhango G, Sigel K, Nicastri DG, Smith CB, Bonomi M, Galsky MD, Taioli E, Neugut AI, Wisnivesky JP. Comparative Effectiveness of Robotic-Assisted Surgery for Resectable Lung Cancer in Older Patients. Chest. 2020 May;157(5):1313-1321. doi: 10.1016/j.chest.2019.09.017. Epub 2019 Oct 4. PMID: 31589843; PMCID: PMC8500998.

Verhagen AF, Schoenmakers MC, Barendregt W, Smit H, van Boven WJ, Looijen M, van der Heijden EH, van Swieten HA. Completeness of lung cancer surgery: is mediastinal dissection common practice? Eur J Cardiothorac Surg. 2012 Apr;41(4):834-8. doi: 10.1093/ejcts/ezr059. Epub 2012 Jan 18. PMID: 22290900.

Vilmann P, Clementsen PF, Colella S, Siemsen M, De Leyn P, Dumonceau JM, Herth FJ, Larghi A, Vazquez-Sequeiros E, Hassan C, Crombag L, Korevaar DA, Konge L, Annema JT. Combined endobronchial and esophageal endosonography for the diagnosis and staging of lung cancer: European Society of Gastrointestinal Endoscopy (ESGE) Guideline, in cooperation with the European Respiratory Society (ERS) and the European Society of Thoracic Surgeons (ESTS). Endoscopy. 2015 Jun;47(6):545-59. doi: 10.1055/s-0034-1392040. Epub 2015 Jun 1. Erratum in: Endoscopy. 2015 Jun;47(6):c1. Vasquez-Sequeiros, Enrique [corrected to Vazquez-Sequeiros, Enrique]. PMID: 26030890.

Wald O, Sadeh BM, Bdolah-Abram T, Erez E, Shapira OM, Izhar U. Outcomes of sublobar resection vs lobectomy for invasive clinical stage T1N0 non-small-cell lung cancer: A propensity-match analysis. Cancer Rep (Hoboken). 2021 Jun;4(3):e1339. doi: 10.1002/cnr2.1339. Epub 2021 Feb 11. PMID: 33570255; PMCID: PMC8222555.

Wang CL, Li Y, Yue DS, Zhang LM, Zhang ZF, Sun BS. Value of the metastatic lymph node ratio for predicting the prognosis of non- small-cell lung cancer patients. World J Surg. 2012 Feb;36(2):455-62. doi: 10.1007/s00268-011-1360-8. PMID: 22187129.

Wang X, Guo H, Hu Q, Ying Y, Chen B. Pulmonary function after segmentectomy versus lobectomy in patients with early-stage non-small- cell lung cancer: a meta-analysis. J Int Med Res. 2021 Sep;49(9):3000605211044204. doi: 10.1177/03000605211044204. PMID: 34521244; PMCID: PMC8447102.

Wang X, Guo H, Hu Q, Ying Y, Chen B. The Impact of Skip vs. Non-Skip N2 Lymph Node Metastasis on the Prognosis of Non-Small-Cell Lung Cancer: A Systematic Review and Meta-Analysis. Front Surg. 2021 Oct 12;8:749156. doi: 10.3389/fsurg.2021.749156. PMID: 34712694; PMCID: PMC8546110.

Wang Z, Yang Z, Li S, Zhang J, Xia L, Zhou J, Chen N, Guo C, Liu L. A Comprehensive Comparison of Different Nodal Subclassification Methods in Surgically Resected Non-Small-Cell Lung Cancer Patients. Ann Surg Oncol. 2022 Dec;29(13):8144-8153. doi: 10.1245/s10434-022-12363-w. Epub 2022 Aug 18. PMID: 35980551.

Watanabe Y, Shimizu J, Oda M, Hayashi Y, Watanabe S, Tatsuzawa Y, Iwa T, Suzuki M, Takashima T. Aggressive surgical intervention in N2 non-small cell cancer of the lung. Ann Thorac Surg. 1991 Feb;51(2):253-61. doi: 10.1016/0003-4975(91)90797-t. PMID: 1846524.

Wei B, Jin X, Lu G, Zhao T, Xue H, Zhang Y. A novel nomogram to predict lymph node metastasis in cT1 non-small-cell lung cancer based on PET/CT and peripheral blood cell parameters. BMC Pulm Med. 2023 Jan 30;23(1):44. doi: 10.1186/s12890-023-02341-7. PMID: 36717907; PMCID: PMC9885665.

Winget M, Stanger J, Gao Z, Butts C. Predictors of surgery and consult with an oncologist for adjuvant chemotherapy in early stage NSCLC patients in Alberta, Canada. J Thorac Oncol. 2009 May;4(5):629-34. doi: 10.1097/JTO.0b013e31819ccf26. PMID: 19276835.

Wisnivesky JP, Arciniega J, Mhango G, Mandeli J, Halm EA. Lymph node ratio as a prognostic factor in elderly patients with pathological N1 non-small cell lung cancer. Thorax. 2011 Apr;66(4):287-93. doi: 10.1136/thx.2010.148601. Epub 2010 Dec 2. PMID: 21131298; PMCID: PMC5642294.

Wozniak AJ, Gadgeel SM. Adjuvant treatment of non-small-cell lung cancer: how do we improve the cure rates further? Oncology (Williston Park). 2007 Feb;21(2):163-71; discussion 171, 174, 179-82. PMID: 17396481.

Wu S, Wang Z, Sun J, Wu H, Jin Z, Ren S, Hu B, Cho WC, Zhu C, Chen T, Shen J. Survival Outcomes of Sublobectomy and Lobectomy in Elderly Patients with Peripheral Solid-Dominant Non-small Cell Lung Cancer. Ann Surg Oncol. 2023 Mar;30(3):1522-1529. doi: 10.1245/s10434-022-12909-y. Epub 2022 Dec 15. PMID: 36520230.

Xie M, Mei X, Li T, Sun X, Xu S, Ma D. [Prognostic analysis of patients with unsuspected pathologic N1 non-small cell lung cancer]. Zhonghua Zhong Liu Za Zhi. 2015 May;37(5):387-91. Chinese. PMID: 26463033.

Xie X, Wang X, Zheng L, Zhang SY, Su XD, Yu H, Li Y, Zhou JL, Ling L, Rong TH. [Value of mediastinoscopy in preoperative staging of non-small cell lung cancer-based on survival analysis]. Zhonghua Zhong Liu Za Zhi. 2009 Dec;31(12):929-32. Chinese. PMID: 20193336.

Yamanashi K, Okumura N, Yamamoto Y, Takahashi A, Nakashima T, Matsuoka T. Comparing Part-Solid and Pure-Solid Tumors in the TNM Classification of Lung Cancer (Eighth Edition). Thorac Cardiovasc Surg. 2019 Jun;67(4):306-314. doi: 10.1055/s-0038-1651521. Epub 2018 May 29. PMID: 29843187.

Yang CF, Adil SM, Anderson KL, Meyerhoff RR, Turley RS, Hartwig MG, Harpole DH Jr, Tong BC, Onaitis MW, D'Amico TA, Berry MF. Impact of patient selection and treatment strategies on outcomes after lobectomy for biopsy-proven stage IIIA pN2 non-small cell lung cancer. Eur J Cardiothorac Surg. 2016 Jun;49(6):1607-13. doi: 10.1093/ejcts/ezv431. Epub 2015 Dec 30. PMID: 26719403; PMCID: PMC4867397.

Yang CJ, Gu L, Shah SA, Yerokun BA, D'Amico TA, Hartwig MG, Berry MF. Long-term outcomes of surgical resection for stage IV non- small-cell lung cancer: A national analysis. Lung Cancer. 2018 Jan;115:75-83. doi: 10.1016/j.lungcan.2017.11.021. Epub 2017 Nov 23. PMID: 29290266.

Yang CJ, Kumar A, Klapper JA, Hartwig MG, Tong BC, Harpole DH Jr, Berry MF, D'Amico TA. A National Analysis of Long-term Survival Following Thoracoscopic Versus Open Lobectomy for Stage I Non-small-cell Lung Cancer. Ann Surg. 2019 Jan;269(1):163-171. doi: 10.1097/SLA.0000000000002342. PMID: 28799982.

Yang CJ, Nwosu A, Mayne NR, Wang YY, Raman V, Meyerhoff RR, D'Amico TA, Berry MF. A Minimally Invasive Approach to Lobectomy After Induction Therapy Does Not Compromise Survival. Ann Thorac Surg. 2020 May;109(5):1503-1511. doi: 10.1016/j.athoracsur.2019.09.065. Epub 2019 Nov 13. PMID: 31733187.

Ye X, Liu Y, Yang J, Wang Y, Cui X, Xie H, Song L, Ding Z, Zhai R, Han Y, Yang L, Zhang H. Do older patients with stage IB non-small-cell lung cancer obtain survival benefits from surgery? A propensity

score matching study using SEER data. Eur J Surg Oncol. 2022 Sep;48(9):1954-1963. doi: 10.1016/j.ejso.2022.03.015. Epub 2022 Mar 21. PMID: 35379544.

Yeh YC, Kadota K, Nitadori J, Sima CS, Rizk NP, Jones DR, Travis WD, Adusumilli PS. International Association for the Study of Lung Cancer/American Thoracic Society/European Respiratory Society classification predicts occult lymph node metastasis in clinically mediastinal node-negative lung adenocarcinoma. Eur J Cardiothorac Surg. 2016 Jan;49(1):e9-e15. doi: 10.1093/ejcts/ezv316. Epub 2015 Sep 15. PMID: 26377636; PMCID: PMC4678972.

Yendamuri S, Dhillon SS, Groman A, Dy G, Dexter E, Picone A, Nwogu C, Demmy T, Hennon M. Effect of the number of lymph nodes examined on the survival of patients with stage I non-small cell lung cancer who undergo sublobar resection. J Thorac Cardiovasc Surg. 2018 Jul;156(1):394-402. doi: 10.1016/j.jtcvs.2018.03.113. Epub 2018 Apr 4. PMID: 29709364.

Yoo C, Yoon S, Lee DH, Park SI, Kim DK, Kim YH, Kim HR, Choi SH, Kim WS, Choi CM, Jang SJ, Song SY, Kim SS, Choi EK, Lee JC, Suh C, Lee JS, Kim SW. Prognostic Significance of the Number of Metastatic pN2 Lymph Nodes in Stage IIIA-N2 Non-Small-Cell Lung Cancer After Curative Resection. Clin Lung Cancer. 2015 Nov;16(6):e203-12. doi: 10.1016/j.cllc.2015.04.004. Epub 2015 Apr 23. PMID: 25997733.

Yoshimura R, Deguchi H, Tomoyasu M, Shigeeda W, Kaneko Y, Iwai H, Saito H. Assessment of lymph node metastasis of ≤20 mm non-small cell lung cancer originating from superior segment compared to basal segment. Thorac Cancer. 2023 Jan;14(3):304-308. doi: 10.1111/1759-7714.14764. Epub 2022 Dec 9. PMID: 36495037; PMCID: PMC9870726.

Yu WS, Shin J, Son JA, Jung J, Haam S. Assessment of textbook outcome after lobectomy for early-stage non-small cell lung cancer in a Korean institution: A retrospective study. Thorac Cancer. 2022 Apr;13(8):1211-1219. doi: 10.1111/1759-7714.14391. Epub 2022 Mar 20. PMID: 35307965; PMCID: PMC9013659.

Yun JK, Bok JS, Lee GD, Kim HR, Kim YH, Kim DK, Park SI, Choi S. Long-term outcomes of upfront surgery in patients with resectable pathological N2 non-small-cell lung cancer. Eur J Cardiothorac Surg. 2020 Jul 1;58(1):59-69. doi: 10.1093/ejcts/ezaa042. PMID: 32155245.

Yun JK, Kwon Y, Kim J, Lee GD, Choi S, Kim HR, Kim YH, Kim DK, Park SI. Clinical impact of histologic type on survival and recurrence in patients with surgically resected stage II and III non-small cell lung cancer. Lung Cancer. 2023 Feb;176:24-30. doi: 10.1016/j.lungcan.2022.12.008. Epub 2022 Dec 22. PMID: 36580727.

Yun JK, Lee GD, Choi S, Kim HR, Kim YH, Kim DK, Park SI. Comparison of prognostic impact of lymphovascular invasion in stage IA non-small cell lung cancer after lobectomy versus sublobar resection: A propensity score-matched analysis. Lung Cancer. 2020 Aug;146:105-111. doi: 10.1016/j.lungcan.2020.04.033. Epub 2020 May 16. PMID: 32526600.

Yun JK, Lee GD, Choi S, Kim HR, Kim YH, Park SI, Kim DK. The addition of radiotherapy to adjuvant chemotherapy has a combinatorial effect in pN2 non-small cell lung cancer only with extranodal invasion or multiple N2 metastasis. Lung Cancer. 2021 May;155:94-102. doi: 10.1016/j.lungcan.2021.03.011. Epub 2021 Mar 16. PMID: 33765654.

Yun JK, Lee GD, Choi S, Kim YH, Kim DK, Park SI, Kim HR. Clinical Significance of Regional Lymph Node Evaluation During Sublobar Resection in Lung Cancer. Ann Thorac Surg. 2022 Sep;114(3):989-997. doi: 10.1016/j.athoracsur.2021.07.095. Epub 2021 Sep 4. PMID: 34487710.

Zemanova M, Pirker R, Petruzelka L, Zbozínkova Z, Jovanovic D, Rajer M, Bogos K, Purkalne G, Ceriman V, Chaudhary S, Richter I, Kufa J, Jakubikova L, Zemaitis M, Cernovska M, Koubkova L, Vilasova Z, Dieckmann K, Farkas A, Spasic J, Fröhlich K, Tiefenbacher A, Hollosi V, Kultan J, Kolarová I, Votruba J. Care of patients with non-small-cell lung cancer stage III - the Central European real-world experience. Radiol Oncol. 2020 May 28;54(2):209-220. doi: 10.2478/raon-2020-0026. PMID: 32463394; PMCID: PMC7276648.

Zhang Y, Liu Z, Wang H, Liang F, Zhu L, Liu H. Association of metastatic nodal size with survival in non-surgical non-small cell lung cancer patients: Recommendations for clinical N staging. Front Oncol. 2022 Oct 21;12:990540. doi: 10.3389/fonc.2022.990540. PMID: 36338722; PMCID: PMC9633939.

Zhang Z, Liu D, Guo Y, Shi B, Song Z, Tian Y. [Effects of multiple factors on the prognosis of pIIIa/N2 patients with non-small cell lung cancer]. Zhongguo Fei Ai Za Zhi. 2010 Aug;13(8):781-5. Chinese. doi: 10.3779/j.issn.1009-3419.2010.08.06. PMID: 20704818; PMCID: PMC6000559.

Zhang ZR, Mao YS, Gao SG, Mu JW, Xue Q, Wang DL, Gao YS, Zhao J, He J. [Survival after surgical treatment of bilateral synchronous multiple primary non-small cell lung cancers]. Zhonghua Zhong Liu Za Zhi. 2016 Jun 23;38(6):460-5. Chinese. doi: 10.3760/cma.j.issn.0253-3766.2016.06.011. PMID: 27346405.

Zheng D, Ye T, Hu H, Zhang Y, Sun Y, Xiang J, Chen H. Upfront surgery as first-line therapy in selected patients with stage IIIA non-small cell lung cancer. J Thorac Cardiovasc Surg. 2018 Apr;155(4):1814-1822.e4. doi: 10.1016/j.jtcvs.2017.10.075. Epub 2017 Nov 3. PMID: 29221745.

Zhong W, Yang X, Bai J, Yang J, Manegold C, Wu Y. Complete mediastinal lymphadenectomy: the core component of the multidisciplinary therapy in resectable non-small cell lung cancer. Eur J Cardiothorac Surg. 2008 Jul;34(1):187-95. doi: 10.1016/j.ejcts.2008.03.060. Epub 2008 May 23. PMID: 18457958.

Zhou B, Zang R, Zhang M, Song P, Liu L, Bie F, Peng Y, Bai G, Gao S. A new N descriptor for non-small cell lung cancer: the classification based on anatomic location, number and ratio of metastatic lymph nodes. Transl Lung Cancer Res. 2022 Aug;11(8):1540-1554. doi: 10.21037/tlcr-21-933. PMID: 36090638; PMCID: PMC9459623.

Zhou S, Pei G, Han Y, Yu D, Song X, Li Y, Xiao N, Liu S, Liu Z, Xu S. Sleeve lobectomy by video-assisted thoracic surgery versus thoracotomy for non-small cell lung cancer. J Cardiothorac Surg. 2015 Sep 10;10:116. doi: 10.1186/s13019-015-0318-6. PMID: 26357875; PMCID: PMC4564953.

Zieliński M, Hauer L, Hauer J, Pankowski J, Szlubowski A, Nabiałek T. TEMLA--rozszerzone wycięcie węzłów chłonnych śródpiersia w diagnostyce niedrobnokomórkowego raka płuca [Transcervical Extended Mediastinal Lymphadenectomy (TEMLA) for staging of non- small-cell lung cancer (NSCLC)]. Pneumonol Alergol Pol. 2011;79(3):196-206. Polish. PMID: 21509732.

Zou JY, Zhao WH, Chen JL, Du XK, Hu XW, Ye ZY. [The role of EBUS-TBNA in the systematic evaluation of lymph node staging and resectability analysis in non-small cell lung cancer]. Zhonghua Zhong Liu Za Zhi. 2019 Oct 23;41(10):792-795. Chinese. doi: 10.3760/cma.j.issn.0253-3766.2019.10.013. PMID: 31648504.

Excluded papers based inclusion of patients with neo-adjuvant therapie

Cerfolio RJ, Ghanim AF, Dylewski M, Veronesi G, Spaggiari L, Park BJ. The long-term survival of robotic lobectomy for non-small cell lung cancer: A multi-institutional study. J Thorac Cardiovasc Surg. 2018 Feb;155(2):778-786. doi: 10.1016/j.jtcvs.2017.09.016. Epub 2017 Sep 18. PMID: 29031947; PMCID: PMC5896345.

Diebels I, Hendriks JMH, Van Meerbeeck JP, Lauwers P, Janssens A, Yogeswaran SK, Van Schil PEY. Evaluation of mediastinoscopy in mediastinal lymph node staging for non-small-cell lung cancer. Interact Cardiovasc Thorac Surg. 2021 Jan 22;32(2):270-275. doi: 10.1093/icvts/ivaa263. PMID: 33257953.

Fiorelli A, Sagan D, Mackiewicz L, Cagini L, Scarnecchia E, Chiodini P, Caronia FP, Puma F, Santini M, Ragusa M. Incidence, Risk Factors, and Analysis of Survival of Unexpected N2 Disease in Stage I Non-Small Cell Lung Cancer. Thorac Cardiovasc Surg. 2015 Oct;63(7):558-67. doi: 10.1055/s-0034-1399764. Epub 2015 Jan 28. PMID: 25629458.

Garelli E, Renaud S, Falcoz PE, Weingertner N, Olland A, Santelmo N, Massard G. Microscopic N2 disease exhibits a better prognosis in resected non-small-cell lung cancer. Eur J Cardiothorac Surg. 2016 Aug;50(2):322-8. doi: 10.1093/ejcts/ezw036. Epub 2016 Feb 25. PMID: 26920941.

Hu XF, Duan L, Jiang GN, Chen C, Fei KE. Surgery following neoadjuvant chemotherapy for non-small-cell lung cancer patients with unexpected persistent pathological N2 disease. Mol Clin Oncol. 2016 Feb;4(2):261-267. doi: 10.3892/mco.2015.706. Epub 2015 Dec 11. PMID: 26893872; PMCID: PMC4734035.

Hwangbo B, Park EY, Yang B, Lee GK, Kim TS, Kim HY, Kim MS, Lee JM. Long-term Survival According to N Stage Diagnosed by Endobronchial Ultrasound-Guided Transbronchial Needle Aspiration in Non-small Cell Lung Cancer. Chest. 2022 May;161(5):1382-1392. doi: 10.1016/j.chest.2021.11.032. Epub 2021 Dec 8. PMID: 34896095.

Inoue M, Sawabata N, Takeda S, Ohta M, Ohno Y, Maeda H. Results of surgical intervention for p-stage IIIA (N2) non-small cell lung cancer: acceptable prognosis predicted by complete resection in patients with single N2 disease with primary tumor in the upper lobe. J Thorac Cardiovasc Surg. 2004 Apr;127(4):1100-6. doi: 10.1016/j.jtcvs.2003.09.012. PMID: 15052208.

Johnson BE, Rabin MS. Patient subsets benefiting from adjuvant therapy following surgical resection of non-small cell lung cancer. Clin Cancer Res. 2005 Jul 1;11(13 Pt 2):5022s-5026s. doi: 10.1158/1078-0432.CCR-05-9001. PMID: 16000607.

Ketchedjian A, Daly BD, Fernando HC, Florin L, Hunter CJ, Morelli DM, Shemin RJ. Location as an important predictor of lymph node involvement for pulmonary adenocarcinoma. J Thorac Cardiovasc Surg. 2006 Sep;132(3):544-8. doi: 10.1016/j.jtcvs.2006.05.023. Epub 2006 Jul 31. PMID: 16935108.

Le Pechoux C, Pourel N, Barlesi F, Lerouge D, Antoni D, Lamezec B, Nestle U, Boisselier P, Dansin E, Paumier A, Peignaux K, Thillays F, Zalcman G, Madelaine J, Pichon E, Larrouy A, Lavole A, Argo-Leignel D, Derollez M, Faivre-Finn C, Hatton MQ, Riesterer O, Bouvier- Morel E, Dunant A, Edwards JG, Thomas PA, Mercier O, Bardet A. Postoperative radiotherapy versus no postoperative radiotherapy in patients with completely resected non-small-cell lung cancer and proven mediastinal N2 involvement (Lung ART): an open-label, randomised, phase 3 trial. Lancet Oncol. 2022 Jan;23(1):104-114. doi: 10.1016/S1470-2045(21)00606-9. Epub 2021 Dec 15. PMID: 34919827.

Moretti L, Yu DS, Chen H, Carbone DP, Johnson DH, Keedy VL, Putnam JB Jr, Sandler AB, Shyr Y, Lu B. Prognostic factors for resected non-small cell lung cancer with pN2 status: implications for use of postoperative radiotherapy. Oncologist. 2009 Nov;14(11):1106-15. doi: 10.1634/theoncologist.2009-0130. Epub 2009 Nov 6. PMID: 19897534; PMCID: PMC3045762.

Navani N, Nankivell M, Stephens RJ, Parmar MK, Gilligan D, Nicolson M, Groen HJ, van Meerbeeck JP. Inaccurate clinical nodal staging of non-small cell lung cancer: evidence from the MRC LU22 multicentre randomised trial. Thorax. 2010 May;65(5):463. doi: 10.1136/thx.2009.118471. PMID: 20435873.

Park SY, Lee HS, Jang HJ, Joo J, Kim MS, Lee JM, Zo JI. Wedge bronchoplastic lobectomy for non-small cell lung cancer as an alternative to sleeve lobectomy. J Thorac Cardiovasc Surg. 2012 Apr;143(4):825-831.e3. doi: 10.1016/j.jtcvs.2011.10.057. Epub 2011 Nov 20. PMID: 22104687.

Schlachtenberger G, Doerr F, Menghesha H, Amorin A, Hoepker K, Hagmeyer L, Wahlers T, Hekmat K, Heldwein MB. Prognostic impact of lymph node spreading pattern in N2 NSCLC patients. Expert Rev Anticancer Ther. 2023 Mar;23(3):319-326. doi: 10.1080/14737140.2023.2174528. Epub 2023 Feb 3. PMID: 36708591.

Shahin GM, Topal B, Pouwels S, Markou TL, Boon R, Stigt JA. Quality assessment of robot assisted thoracic surgical resection of non- small cell lung cancer: nodal upstaging and mediastinal recurrence. J Thorac Dis. 2021 Feb;13(2):592-599. doi: 10.21037/jtd-20-2267. PMID: 33717532; PMCID: PMC7947478.

Shahin GMM, Vos PWK, Hutteman M, Stigt JA, Braun J. Robot-assisted thoracic surgery for stages IIB-IVA non-small cell lung cancer: retrospective study of feasibility and outcome. J Robot Surg. 2023 Mar 16. doi: 10.1007/s11701-023-01549-3. Epub ahead of print. PMID: 36928749.

Shapiro M, Kadakia S, Lim J, Breglio A, Wisnivesky JP, Kaufman A, Lee DS, Flores RM. Lobe-specific mediastinal nodal dissection is sufficient during lobectomy by video-assisted thoracic surgery or thoracotomy for early-stage lung cancer. Chest. 2013 Nov;144(5):1615- 1621. doi: 10.1378/chest.12-3069. PMID: 23828253.

Sonobe M, Date H, Wada H, Okubo K, Hamakawa H, Teramukai S, Matsumura A, Nakagawa T, Sumitomo S, Miyamoto Y, Okumura N, Takeo S, Kawakami K, Aoki M, Kosaka S; The Japan-Multinational Trial Organization. Prognostic factors after complete resection of pN2 non-small cell lung cancer. J Thorac Cardiovasc Surg. 2013 Oct;146(4):788-95. doi: 10.1016/j.jtcvs.2013.04.043. Epub 2013 Jun 27. PMID: 23810113.

Stamatis G, Müller S, Weinreich G, Schwarz B, Eberhardt W, Pöttgen C, Aigner C. Significantly favourable outcome for patients with non-small-cell lung cancer stage IIIA/IIIB and single-station persistent N2 (skip or additionally N1) disease after multimodality treatment. Eur J Cardiothorac Surg. 2022 Jan 24;61(2):269-276. doi: 10.1093/ejcts/ezab372. PMID: 34368849.

Tanaka S, Aoki M, Ishikawa H, Otake Y. Pneumonectomy for node-positive non-small cell lung cancer: can it be a treatment option for N2 disease? Gen Thorac Cardiovasc Surg. 2014 Jun;62(6):370-5. doi: 10.1007/s11748-014-0380-3. Epub 2014 Mar 1. PMID: 24578122.

Taylor MD, Nagji AS, Bhamidipati CM, Theodosakis N, Kozower BD, Lau CL, Jones DR. Tumor recurrence after complete resection for non-small cell lung cancer. Ann Thorac Surg. 2012 Jun;93(6):1813-20; discussion 1820-1. doi: 10.1016/j.athoracsur.2012.03.031. Epub 2012 Apr 26. PMID: 22542070.

Tsitsias T, Okiror L, Veres L, King J, Harrison-Phipps K, Routledge T, Pilling J, Bille A. New N1/N2 classification and lobe specific lymphatic drainage: Impact on survival in patients with non-small cell lung cancer treated with surgery. Lung Cancer. 2021 Jan;151:84-90. doi: 10.1016/j.lungcan.2020.11.005. Epub 2020 Nov 16. PMID: 33250210.

Veronesi G, Park B, Cerfolio R, Dylewski M, Toker A, Fontaine JP, Hanna WC, Morenghi E, Novellis P, Velez-Cubian FO, Amaral MH, Dieci E, Alloisio M, Toloza EM. Robotic resection of Stage III lung cancer: an international retrospective study. Eur J Cardiothorac Surg. 2018 Nov 1;54(5):912-919. doi: 10.1093/ejcts/ezy166. PMID: 29718155; PMCID: PMC6454562.

Watanabe Y, Hayashi Y, Takabatake I, Shimizu J, Murakami S, Morita K, Arano Y, Nonomura A. [Clinical significance of extended mediastinal lymph node dissection on the basis of clinicopathological analysis of nodal involvement in bronchogenic carcinoma]. Kyobu Geka. 1994 Jan;47(1):4-9. Japanese. PMID: 8277631.

Yang H, Dai L, Li P, Shen L, Yan W, Fan M, Chen K. [Survival Analysis of 121 Stage N2-IIIa Non-small Cell Lung Cancer Patients Treated with Surgery]. Zhongguo Fei Ai Za Zhi. 2015 Aug;18(8):505-11. Chinese. doi: 10.3779/j.issn.1009-3419.2015.08.06. PMID: 26302348; PMCID: PMC6000234.

Excluded papers based on inadequate or unknown mediastinal staging

Abrão FC, de Abreu IRLB, Silva VG, Rosamilia GA, Peres SV, Hanriot RM, Younes RN. Overall survival and prognostic factors in Stage I lung adenocarcinoma treated with curative intent: A real-life 19-year cohort study. J Surg Oncol. 2022 Nov;126(6):1114-1122. doi: 10.1002/jso.27015. Epub 2022 Jul 18. PMID: 35848402.

Abughararah TZ, Jeong YH, Alabbood F, Chong Y, Yun JK, Lee GD, Choi S, Kim HR, Kim YH, Kim DK, Park SI. Lobe-specific lymph node dissection in stage IA non-small-cell lung cancer: a retrospective cohort study. Eur J Cardiothorac Surg. 2021 Apr 29;59(4):783-790. doi: 10.1093/ejcts/ezaa369. PMID: 33150427.

Ajmani GS, Wang CH, Kim KW, Howington JA, Krantz SB. Surgical quality of wedge resection affects overall survival in patients with early stage non-small cell lung cancer. J Thorac Cardiovasc Surg. 2018 Jul;156(1):380-391.e2. doi: 10.1016/j.jtcvs.2018.02.095. Epub 2018 Mar 13. PMID: 29680711.

Akçay, O., 2017. Skip metastasis in non-small cell lung cancer: does it affect the prognosis?. The Turkish Journal of Thoracic and Cardiovascular Surgery, 25(2), pp.230-234.

Al-Sarraf N, Aziz R, Gately K, Lucey J, Wilson L, McGovern E, Young V. Pattern and predictors of occult mediastinal lymph node involvement in non-small cell lung cancer patients with negative mediastinal uptake on positron emission tomography. Eur J Cardiothorac Surg. 2008 Jan;33(1):104-9. doi: 10.1016/j.ejcts.2007.09.026. Epub 2007 Oct 30. PMID: 17977738.

Andersson S, Ilonen I, Järvinen T, Rauma V, Räsänen J, Salo J. Surgically Treated Unsuspected N2-Positive NSCLC: Role of Extent and Location of Lymph Node Metastasis. Clin Lung Cancer. 2018 Sep;19(5):418-425. doi: 10.1016/j.cllc.2018.04.011. Epub 2018 May 5. PMID: 29880414.

Andre F, Grunenwald D, Pignon JP, Dujon A, Pujol JL, Brichon PY, Brouchet L, Quoix E, Westeel V, Le Chevalier T. Survival of patients with resected N2 non-small-cell lung cancer: evidence for a subclassification and implications. J Clin Oncol. 2000 Aug;18(16):2981-9. doi: 10.1200/JCO.2000.18.16.2981. PMID: 10944131.

Ayub A, Rehmani SS, Al-Ayoubi AM, Raad W, Flores RM, Bhora FY. Pulmonary Resection for Second Lung Cancer After Pneumonectomy: A Population-Based Study. Ann Thorac Surg. 2017 Oct;104(4):1131-1137. doi: 10.1016/j.athoracsur.2017.04.043. Epub 2017 Jul 12. PMID: 28709663.

Baba T, Uramoto H, Kuwata T, Chikaishi Y, Nakagawa M, So T, Hanagiri T, Tanaka F. Survival impact of node zone classification in resected pathological N2 non-small cell lung cancer. Interact Cardiovasc Thorac Surg. 2012 Jun;14(6):760-4. doi: 10.1093/icvts/ivs058. Epub 2012 Feb 27. PMID: 22374294; PMCID: PMC3352724.

Bertoglio P, Ricciardi S, Alì G, Aprile V, Korasidis S, Palmiero G, Fontanini G, Mussi A, Lucchi M. N2 lung cancer is not all the same: an analysis of different prognostic groups. Interact Cardiovasc Thorac Surg. 2018 Nov 1;27(5):720-726. doi: 10.1093/icvts/ivy171. PMID: 29788107.

Beyaz F, Verhoeven RLJ, Schuurbiers OCJ, Verhagen AFTM, van der Heijden EHFM. Occult lymph node metastases in clinical N0/N1 NSCLC; A single center in-depth analysis. Lung Cancer. 2020 Dec;150:186-194. doi: 10.1016/j.lungcan.2020.10.022. Epub 2020 Nov 7. PMID: 33189983.

Bousema JE, Aarts MJ, Dijkgraaf MGW, Annema JT, van den Broek FJC. Trends in mediastinal nodal staging and its impact on unforeseen N2 and survival in lung cancer. Eur Respir J. 2021 Apr 1;57(4):2001549. doi: 10.1183/13993003.01549-2020. PMID: 33008940.

Cangemi V, Volpino P, D'Andrea N, Chiarotti F, Tomassini R, Piat G. Results of surgical treatment of stage IIIA non-small cell lung cancer. Eur J Cardiothorac Surg. 1995;9(7):352-9. doi: 10.1016/s1010-7940(05)80167-8. PMID: 8519514.

Cerfolio RJ, Bryant AS. Survival of patients with unsuspected N2 (stage IIIA) nonsmall-cell lung cancer. Ann Thorac Surg. 2008 Aug;86(2):362-6; discussion 366-7. doi: 10.1016/j.athoracsur.2008.04.042. PMID: 18640297.

Ceylan N, Doğan S, Kocaçelebi K, Savaş R, Çakan A, Çağrici U. Contrast enhanced CT versus integrated PET-CT in pre-operative nodal staging of non-small cell lung cancer. Diagn Interv Radiol. 2012 Sep-Oct;18(5):435-40. doi: 10.4261/1305-3825.DIR.5100-11.2. Epub 2012 Feb 29. PMID: 22374706.

Chella A, Lucchi M, Gragnani F, Ribechini A, Silvano G, Janni A, Mussi A, Angeletti CA. Ruolo delle terapie adiuvanti nel trattamento del carcinoma polmonare non a piccole cellule pN2 [The role of adjuvant therapy in the management of pN2 non-small-cell carcinoma of the lung]. Minerva Chir. 1995 Dec;50(12):1029-38. Italian. PMID: 8725059.

Chen JX, Lu TY, Lin YS, Fang HY, Shih PK. Prognostic effect of incongruous lymph node status in early-stage non-small cell lung cancer. Eur J Surg Oncol. 2021 Feb;47(2):450-455. doi: 10.1016/j.ejso.2020.06.003. Epub 2020 Jun 10. PMID: 32928610.

Chen W, Zhang C, Wang G, Yu Z, Liu H. Feasibility of nodal classification for non-small cell lung cancer by merging current N categories with the number of involved lymph node stations. Thorac Cancer. 2019 Jul;10(7):1533-1543. doi: 10.1111/1759-7714.13094. Epub 2019 Jun 17. PMID: 31207184; PMCID: PMC6610263.

Chiappetta M, Leuzzi G, Sperduti I, Bria E, Mucilli F, Lococo F, Spaggiari L, Ratto GB, Filosso PL, Facciolo F. Lymph-node ratio predicts survival among the different stages of non-small-cell lung cancer: a multicentre analysis†. Eur J Cardiothorac Surg. 2019 Mar 1;55(3):405- 412. doi: 10.1093/ejcts/ezy311. PMID: 30202953.

Chiappetta M, Lococo F, Leuzzi G, Sperduti I, Bria E, Petracca Ciavarella L, Mucilli F, Filosso PL, Ratto G, Spaggiari L, Facciolo F, Margaritora S. Survival Analysis in Single N2 Station Lung Adenocarcinoma: The Prognostic Role of Involved Lymph Nodes and Adjuvant Therapy. Cancers (Basel). 2021 Mar 16;13(6):1326. doi: 10.3390/cancers13061326. PMID: 33809513; PMCID: PMC7998125.

Chiappetta M, Lococo F, Leuzzi G, Sperduti I, Petracca-Ciavarella L, Bria E, Mucilli F, Filosso PL, Ratto GB, Spaggiari L, Facciolo F, Margaritora S. External validation of the N descriptor in the proposed tumour-node-metastasis subclassification for lung cancer: the crucial role of histological type, number of resected nodes and adjuvant therapy. Eur J Cardiothorac Surg. 2020 Dec 1;58(6):1236-1244. doi: 10.1093/ejcts/ezaa215. PMID: 32770184.

Cho HJ, Kim SR, Kim HR, Han JO, Kim YH, Kim DK, Park SI. Modern outcome and risk analysis of surgically resected occult N2 non- small cell lung cancer. Ann Thorac Surg. 2014 Jun;97(6):1920-5. doi: 10.1016/j.athoracsur.2014.03.004. Epub 2014 Apr 24. PMID: 24768044.

Cruz C, Afonso M, Oliveiros B, Pêgo A. Recurrence and Risk Factors for Relapse in Patients with Non-Small Cell Lung Cancer Treated by Surgery with Curative Intent. Oncology. 2017;92(6):347-352. doi: 10.1159/000458533. Epub 2017 Mar 10. PMID: 28278499.

Darling GE, Allen MS, Decker PA, Ballman K, Malthaner RA, Inculet RI, Jones DR, McKenna RJ, Landreneau RJ, Rusch VW, Putnam JB Jr. Randomized trial of mediastinal lymph node sampling versus complete lymphadenectomy during pulmonary resection in the patient with N0 or N1 (less

than hilar) non-small cell carcinoma: results of the American College of Surgery Oncology Group Z0030 Trial. J Thorac Cardiovasc Surg. 2011 Mar;141(3):662-70. doi: 10.1016/j.jtcvs.2010.11.008. PMID: 21335122; PMCID: PMC5082844.

De Leyn P, Schoonooghe P, Deneffe G, Van Raemdonck D, Coosemans W, Vansteenkiste J, Lerut T. Surgery for non-small cell lung cancer with unsuspected metastasis to ipsilateral mediastinal or subcarinal nodes (N2 disease). Eur J Cardiothorac Surg. 1996;10(8):649-54; discussion 654-5. doi: 10.1016/s1010-7940(96)80380-0. PMID: 8875173.

Defranchi SA, Cassivi SD, Nichols FC, Allen MS, Shen KR, Deschamps C, Wigle DA. N2 disease in T1 non-small cell lung cancer. Ann Thorac Surg. 2009 Sep;88(3):924-8. doi: 10.1016/j.athoracsur.2009.05.039. PMID: 19699921; PMCID: PMC2930772.

Deng HY, Zhou J, Wang RL, Jiang R, Zhu DX, Tang XJ, Zhou Q. Lobe-Specific Lymph Node Dissection for Clinical Early-Stage (cIA) Peripheral Non-small Cell Lung Cancer Patients: What and How? Ann Surg Oncol. 2020 Feb;27(2):472-480. doi: 10.1245/s10434-019- 07926-3. Epub 2019 Oct 15. PMID: 31617120.

DuComb EA, Tonelli BA, Tuo Y, Cole BF, Mori V, Bates JHT, Washko GR, San José Estépar R, Kinsey CM. Evidence for Expanding Invasive Mediastinal Staging for Peripheral T1 Lung Tumors. Chest. 2020 Nov;158(5):2192-2199. doi: 10.1016/j.chest.2020.05.607. Epub 2020 Jun 26. PMID: 32599066; PMCID: PMC8173766.

Dziedzic D, Rudzinski P, Langfort R, Orlowski T; Polish Lung Cancer Study Group (PLCSG). Results of surgical treatment and impact on T staging of non-small-cell lung cancer adjacent lobe invasion. Eur J Cardiothorac Surg. 2016 Sep;50(3):423-7. doi: 10.1093/ejcts/ezw110. Epub 2016 Mar 31. PMID: 27032471.

Eckardt J, Jakobsen E, Licht PB. Subcarinal Lymph Nodes Should be Dissected in All Lobectomies for Non-Small Cell Lung Cancer- Regardless of Primary Tumor Location. Ann Thorac Surg. 2017 Apr;103(4):1121-1125. doi: 10.1016/j.athoracsur.2016.09.109. Epub 2017 Jan 18. PMID: 28109572.

Erdoğu V, Çıtak N, Sezen CB, Kizir D, Tanrıkulu G, Doğru MV, Seyrek Y, Cansever L, Saydam Ö, Metin M. Survival impact of unexpected N2 in stage IIIB/N2 non-small cell lung cancer patients. Asian Cardiovasc Thorac Ann. 2023 Mar;31(3):238-243. doi: 10.1177/02184923231151503. Epub 2023 Jan 22. PMID: 36683332.

Fang C, Xiang Y, Han W. Preoperative risk factors of lymph node metastasis in clinical N0 lung adenocarcinoma of 3 cm or less in diameter. BMC Surg. 2022 Apr 29;22(1):153. doi: 10.1186/s12893-022-01605-z. PMID: 35488235; PMCID: PMC9052540.

Fiorelli A, Caronia FP, Daddi N, Loizzi D, Ampollini L, Ardò N, Ventura L, Carbognani P, Potenza R, Ardissone F, Sollitto F, Mattioli S, Puma F, Santini M, Ragusa M. Sublobar resection versus lobectomy for stage I non-small cell lung cancer: an appropriate choice in elderly patients? Surg Today. 2016 Dec;46(12):1370-1382. doi: 10.1007/s00595-016-1314-8. Epub 2016 Apr 16. PMID: 27085869.

Fukuse T, Hirata T, Naiki H, Hitomi S, Wada H. Prognostic significance of proliferative activity in pN2 non-small-cell lung carcinomas and their mediastinal lymph node metastases. Ann Surg. 2000 Jul;232(1):112-8. doi: 10.1097/00000658-200007000-00016. PMID: 10862203; PMCID: PMC1421115.

Ginsberg RJ, Rubinstein LV. Randomized trial of lobectomy versus limited resection for T1 N0 non-small cell lung cancer. Lung Cancer Study Group. Ann Thorac Surg. 1995 Sep;60(3):615-22; discussion 622-3. doi: 10.1016/0003-4975(95)00537-u. PMID: 7677489.

Goldstraw P, Mannam GC, Kaplan DK, Michail P. Surgical management of non-small-cell lung cancer with ipsilateral mediastinal node metastasis (N2 disease). J Thorac Cardiovasc Surg. 1994 Jan;107(1):19-27; discussion 27-8. PMID: 8283883.

Grodzki T, Alchimowicz J, Kozak A, Kubisa B, Pieróg J, Wójcik J, Bielewicz M, Witkowska D. Additional pulmonary resections after pneumonectomy: actual long-term survival and functional results. Eur J Cardiothorac Surg. 2008 Sep;34(3):493-8. doi: 10.1016/j.ejcts.2008.05.023. Epub 2008 Jun 25. PMID: 18583143.

Haque W, Singh A, Park HS, Teh BS, Butler EB, Zeng M, Lin SH, Welsh JW, Chang JY, Verma V. Quantifying the rate and predictors of occult lymph node involvement in patients with clinically node-negative non-small cell lung cancer. Acta Oncol. 2022 Apr;61(4):403-408. doi: 10.1080/0284186X.2021.2012253. Epub 2021 Dec 16. PMID: 34913815.

Hishida T, Yoshida J, Nishimura M, Nishiwaki Y, Nagai K. Problems in the current diagnostic standards of clinical N1 non-small cell lung cancer. Thorax. 2008 Jun;63(6):526-31. doi: 10.1136/thx.2006.062760. Epub 2007 Nov 16. PMID: 18024539.

Ilhan M, Demir A, Akin H, Zek Gunluoglu M, Olçmen A, Dncer SI. Characteristics and prognosis of resected T3 non-small cell lung cancer. Minerva Chir. 2008 Apr;63(2):101-8. PMID: 18427442.

Ilic N, Petricevic A, Arar D, Kotarac S, Banovic J, Ilic NF, Tripkovic A, Grandic L. Skip mediastinal nodal metastases in the IIIa/N2 non- small cell lung cancer. J Thorac Oncol. 2007 Nov;2(11):1018-21. doi: 10.1097/JTO.0b013e318158d471. PMID: 17975493.

Isaka M, Kojima H, Takahashi S, Omae K, Ohde Y. Risk factors for local recurrence after lobectomy and lymph node dissection in patients with non-small cell lung cancer: Implications for adjuvant therapy. Lung Cancer. 2018 Jan;115:28-33. doi: 10.1016/j.lungcan.2017.11.014. Epub 2017 Nov 16. PMID: 29290258.

Jeon HW, Moon MH, Kim KS, Kim YD, Wang YP, Park HJ, Park JK. Extent of removal for mediastinal nodal stations for patients with clinical stage I non-small cell lung cancer: effect on outcome. Thorac Cardiovasc Surg. 2014 Oct;62(7):599-604. doi: 10.1055/s-0033- 1360478. Epub 2014 Jan 13. PMID: 24420680.

Jin J, Xu Y, Hu X, Chen M, Fang M, Hang Q, Chen M. Postoperative radiotherapy option based on mediastinal lymph node reclassification for patients with pN2 non-small-cell lung cancer. Curr Oncol. 2020 Jun;27(3):e283-e293. doi: 10.3747/co.27.5899. Epub 2020 Jun 1. PMID: 32669935; PMCID: PMC7339838.

Joshi V, McShane J, Page R, Carr M, Mediratta N, Shackcloth M, Poullis M. Clinical upstaging of non-small cell lung cancer that extends across the fissure: implications for non-small cell lung cancer staging. Ann Thorac Surg. 2011 Feb;91(2):350-3. doi: 10.1016/j.athoracsur.2010.09.075. PMID: 21256266.

Kamigaichi A, Aokage K, Katsumata S, Ishii G, Wakabayashi M, Miyoshi T, Tane K, Samejima J, Tsuboi M. Prognostic impact of examined mediastinal lymph node count in clinical N0 non-small cell lung cancer. Eur J Cardiothorac Surg. 2022 Jul 11;62(2):ezac359. doi: 10.1093/ejcts/ezac359. PMID: 35781338.

Kanzaki R, Ikeda N, Okura E, Kitahara N, Shintani Y, Okimura A, Kawahara K, Ohta M. Surgical results and staging of non-small cell lung cancer with interlobar pleural invasion. Interact Cardiovasc Thorac Surg. 2012 Jun;14(6):739-42. doi: 10.1093/icvts/ivs094. Epub 2012 Mar 14. PMID: 22422874; PMCID: PMC3352743.

Kawamoto N, Tsutani Y, Kamigaichi A, Ohsawa M, Mimae T, Miyata Y, Okada M. Tumour location predicts occult N1 nodal metastasis in clinical stage I non-small-cell lung cancer. Eur J Cardiothorac Surg. 2023 Feb 3;63(2):ezac575. doi: 10.1093/ejcts/ezac575. PMID: 36571485.

Kawasaki K, Sato Y, Suzuki Y, Saito H, Nomura Y, Yoshida Y. Prognostic Factors for Surgically Resected N2 Non-small Cell Lung Cancer. Ann Thorac Cardiovasc Surg. 2015;21(3):217-22. doi: 10.5761/atcs.oa.14-00218. Epub 2015 Jan 26. PMID: 25641029; PMCID: PMC4989966.

Keller SM, Vangel MG, Wagner H, Schiller JH, Herskovic A, Komaki R, Marks RS, Perry MC, Livingston RB, Johnson DH; Eastern Cooperative Oncology Group. Prolonged survival in patients with resected non-small cell lung cancer and single-level N2 disease. J Thorac Cardiovasc Surg. 2004 Jul;128(1):130-7. doi: 10.1016/j.jtcvs.2003.11.061. PMID: 15224032.

Kent M, Landreneau R, Mandrekar S, Hillman S, Nichols F, Jones D, Starnes S, Tan A, Putnam J, Meyers B, Daly B, Fernando HC. Segmentectomy versus wedge resection for non-small cell lung cancer in high-risk operable patients. Ann Thorac Surg. 2013 Nov;96(5):1747-54; discussion 1754-5. doi: 10.1016/j.athoracsur.2013.05.104. Epub 2013 Aug 30. PMID: 23998400.

Khullar OV, Liu Y, Gillespie T, Higgins KA, Ramalingam S, Lipscomb J, Fernandez FG. Survival After Sublobar Resection versus Lobectomy for Clinical Stage IA Lung Cancer: An Analysis from the National Cancer Data Base. J Thorac Oncol. 2015 Nov;10(11):1625- 33. doi: 10.1097/JTO.0000000000000664. PMID: 26352534; PMCID: PMC5798611.

Kneuertz PJ, Cheufou DH, D'Souza DM, Mardanzai K, Abdel-Rasoul M, Theegarten D, Moffatt-Bruce SD, Aigner C, Merritt RE. Propensity-score adjusted comparison of pathologic nodal upstaging by robotic, video-assisted thoracoscopic, and open lobectomy for non- small cell lung cancer. J Thorac Cardiovasc Surg. 2019 Nov;158(5):1457-1466.e2. doi: 10.1016/j.jtcvs.2019.06.113. Epub 2019 Aug 28. PMID: 31623811.

Koike T, Nakamura A, Shimizu Y, Goto T, Sato S, Toyabe SI, Tsuchida M. Characteristics and risk factors of recurrence in clinical stage I non-small cell lung cancer patients undergoing anatomic segmentectomy. Gen Thorac Cardiovasc Surg. 2020 Sep;68(9):1011-1017. doi: 10.1007/s11748-020-01338-x. Epub 2020 Mar 20. PMID: 32198710.

Krdzalic G, Mesic D, Iljazovic E, Brkic S, Krdzalic A, Ramic N, Aljic Z, Musanovic N. Mediastinal lymph node metastasis pattern in clinically N0 non-small-cell lung cancer patients who underwent surgical resection. Med Arh. 2010;64(6):332-4. PMID: 21218749.

Kumar A, Deng JZ, Raman V, Okusanya OT, Baiu I, Berry MF, D'Amico TA, Yang CJ. A National Analysis of Minimally Invasive Vs Open Segmentectomy for Stage IA Non-Small-Cell Lung Cancer. Semin Thorac Cardiovasc Surg. 2021 Summer;33(2):535-544. doi: 10.1053/j.semtcvs.2020.09.009. Epub 2020 Sep 23. PMID: 32977013.

Lee DH, Kim JB, Keum DY, Hwang I, Park CK. Long term survival of patients with unsuspected n2 disease in non-small cell lung cancer. Korean J Thorac Cardiovasc Surg. 2013 Feb;46(1):49-55. doi: 10.5090/kjtcs.2013.46.1.49. Epub 2013 Feb 6. PMID: 23423241; PMCID: PMC3573165.

Lee JG, Lee CY, Bae MK, Park IK, Kim DJ, Kim KD, Chung KY. Validity of International Association for the Study Of Lung Cancer proposals for the revision of N descriptors in lung cancer. J Thorac Oncol. 2008 Dec;3(12):1421-6. doi: 10.1097/JTO.0b013e31818e0dbd. PMID: 19057267.

Lee JG, Lee CY, Park IK, Kim DJ, Cho SH, Kim KD, Chung KY. The prognostic significance of multiple station N2 in patients with surgically resected stage IIIA N2 non-small cell lung cancer. J Korean Med Sci. 2008 Aug;23(4):604-8. doi: 10.3346/jkms.2008.23.4.604. PMID: 18756045; PMCID: PMC2526397.

Legras A, Mordant P, Arame A, Foucault C, Dujon A, Le Pimpec Barthes F, Riquet M. Long-term survival of patients with pN2 lung cancer according to the pattern of lymphatic spread. Ann Thorac Surg. 2014 Apr;97(4):1156-62. doi: 10.1016/j.athoracsur.2013.12.047. Epub 2014 Feb 26. PMID: 24582052.

Li FW, Jiang GC, Li Y, Bu L, Yang F, Li JF, Zhao H, Liu YG, Zhou ZL, Liu J, Wang J. [Preliminary comparison research of thoracoscopy and thoracotomy lobectomy for clinical N0 and post-operatively pathological N2 non-small cell lung cancer]. Beijing Da Xue Xue Bao Yi Xue Ban. 2011 Dec 18;43(6):861-5. Chinese. PMID: 22178835.

Li H, Wang R, Zhang D, Zhang Y, Li W, Zhang B, Liu Q, Du J. Lymph node metastasis outside of a tumor-bearing lobe in primary lung cancer and the status of interlobar fissures: The necessity for removing lymph nodes from an adjacent lobe. Medicine (Baltimore). 2019 Mar;98(12):e14800. doi: 10.1097/MD.0000000000014800. PMID: 30896623; PMCID: PMC6709091.

Li X, Huang K, Deng H, Zheng Q, Xiao T, Yu J, Zhou Q. Feasibility and oncological outcomes of video-assisted thoracic surgery versus thoracotomy for pathologic N2 disease in non-small cell lung cancer: A comprehensive systematic review and meta-analysis. Thorac Cancer. 2022 Nov;13(21):2917-2928. doi: 10.1111/1759-7714.14614. Epub 2022 Sep 14. PMID: 36102196; PMCID: PMC9626309.

Li X, Li X, Fu X, Liu L, Liu Y, Zhao H, Li Y, Hu J, Xu L, Liu D, Yang H, Zhang X. Survival benefit of skip metastases in surgically resected N2 non-small cell lung cancer: A multicenter observational study of a large cohort of the Chinese patients. Eur J Surg Oncol. 2020 Oct;46(10 Pt A):1874-1881. doi: 10.1016/j.ejso.2019.12.015. Epub 2019 Dec 18. PMID: 31902592.

Li Y, Wang J. Comparison of clinical outcomes for patients with clinical N0 and pathologic N2 non-small cell lung cancer after thoracoscopic lobectomy and open lobectomy: a retrospective analysis of 76 patients. J Surg Oncol. 2012 Sep 15;106(4):431-5. doi: 10.1002/jso.23104. Epub 2012 Mar 22. PMID: 22442010.

Li ZX, Yang H, She KL, Zhang MX, Xie HQ, Lin P, Zhang LJ, Li XD. The role of segmental nodes in the pathological staging of non-small cell lung cancer. J Cardiothorac Surg. 2013 Dec 8;8:225. doi: 10.1186/1749-8090-8-225. PMID: 24314101; PMCID: PMC4028805.

Liou DZ, Chan M, Bhandari P, Lui NS, Backhus LM, Shrager JB, Berry MF. Lobar versus sublobar resection in clinical stage IA primary lung cancer with occult N2 disease. Eur J Cardiothorac Surg. 2022 Oct 4;62(5):ezac440. doi: 10.1093/ejcts/ezac440. PMID: 36063054.

Liu JW, Li J, Lin G, Shang XQ. [Prognostic analysis of curative surgery for stage IIIA-N2 non-small cell lung cancer]. Zhonghua Zhong Liu Za Zhi. 2013 Jan;35(1):50-3. Chinese. doi: 10.3760/cma.j.issn.0253-3766.2013.01.011. PMID: 23648301.

Liu K, Chen HL, You QS, Huang JF, Wang H. [Clinical analysis of skip N2 metastases in stage IIIA non-small cell lung cancer]. Ai Zheng. 2009 Jul;28(7):725-9. Chinese. doi: 10.5732/cjc.008.10849. PMID: 19624899.

Luan TMB, Bang HT, Vuong NL, Dung LT, Tin NT, Tien TQ, Nam NH. Long-term outcomes of video-assisted lobectomy in non-small cell lung cancer. Asian Cardiovasc Thorac Ann. 2021 May;29(4):318-326. doi: 10.1177/0218492321997380. Epub 2021 Feb 25. PMID: 33631956.

Lutfi W, Schuchert MJ, Dhupar R, Ekeke C, Sarkaria IS, Christie NA, Luketich JD, Okusanya OT. Node-Positive Segmentectomy for Non- Small-Cell Lung Cancer: Risk Factors and Outcomes. Clin Lung Cancer. 2019 Jul;20(4):e463-e469. doi: 10.1016/j.cllc.2019.03.006. Epub 2019 Apr 1. PMID: 31031205; PMCID: PMC8669738.

Maniwa T, Kimura T, Ohue M, Okami J. Mediastinal lymph node dissection in older patients with non-small cell lung cancer. Surg Today. 2022 Mar;52(3):458-464. doi: 10.1007/s00595-021-02373-8. Epub 2021 Sep 15. PMID: 34524511.

Maniwa T, Kimura T, Ohue M, Shintani Y, Okami J. Non-adjacent interlobar lymph node metastasis distant from small-sized peripheral non-small cell lung cancer. Surg Today. 2022 Dec;52(12):1746-1752. doi: 10.1007/s00595-022-02507-6. Epub 2022 Apr 30. PMID: 35501495.

Marulli G, Faccioli E, Mammana M, Nicotra S, Comacchio G, Verderi E, De Palma A, Rea F; Italian VATS Group. Predictors of nodal upstaging in patients with cT1-3N0 non-small cell lung cancer (NSCLC): results from the Italian VATS Group Registry. Surg Today. 2020 Jul;50(7):711-718. doi: 10.1007/s00595-019-01939-x. Epub 2019 Dec 23. Erratum in: Surg Today. 2020 May 10;: PMID: 31873770.

Matsuguma H, Oki I, Nakahara R, Ohata N, Igarashi S, Mori K, Endo S, Yokoi K. Proposal of new nodal classifications for non-small-cell lung cancer based on the number and ratio of metastatic lymph nodes. Eur J Cardiothorac Surg. 2012 Jan;41(1):19-24. doi: 10.1016/j.ejcts.2011.04.016. PMID: 21620720; PMCID: PMC3241096.

Matsuura Y, Ichinose J, Nakao M, Ninomiya H, Nishio M, Okumura S, Mun M. Outcomes of nodal upstaging comparing video-assisted thoracoscopic surgery versus open thoracotomy for lung cancer. Lung Cancer. 2021 Feb;152:78-85. doi: 10.1016/j.lungcan.2020.12.017. Epub 2020 Dec 20. PMID: 33360439.

McPherson I, Bradley NA, Govindraj R, Kennedy ED, Kirk AJB, Asif M. The progression of non-small cell lung cancer from diagnosis to surgery. Eur J Surg Oncol. 2020 Oct;46(10 Pt A):1882-1887. doi: 10.1016/j.ejso.2020.08.013. Epub 2020 Aug 15. PMID: 32847696.

Merritt RE, Abdel-Rasoul M, D'Souza DM, Kneuertz PJ. Lymph Node Upstaging for Robotic, Thoracoscopic, and Open Lobectomy for Stage T2-3N0 Lung Cancer. Ann Thorac Surg. 2023 Jan;115(1):175-182. doi: 10.1016/j.athoracsur.2022.05.041. Epub 2022 Jun 14. PMID: 35714729.

Miyamoto H, Wang Z, Fukai R, Futagawa T, Anami Y, Yamazaki A, Morio A, Hata E. Complete resection via medial sternotomy for non- small cell lung cancer in the right upper lobe. ANZ J Surg. 2005 Dec;75(12):1049-54. doi: 10.1111/j.1445-2197.2005.03614.x. PMID: 16398809.

Miyasaka Y, Suzuki K, Takamochi K, Matsunaga T, Oh S. The maximum standardized uptake value of fluorodeoxyglucose positron emission tomography of the primary tumour is a good predictor of pathological nodal involvement in clinical N0 non-small-cell lung cancer. Eur J Cardiothorac Surg. 2013 Jul;44(1):83-7. doi: 10.1093/ejcts/ezs604. Epub 2012 Dec 11. PMID: 23233074.

Mizuno T, Arimura T, Kuroda H, Sakakura N, Yatabe Y, Sakao Y. Histological type predicts mediastinal metastasis and surgical outcome in resected cN1 non-small cell lung cancer. Gen Thorac Cardiovasc Surg. 2017 Sep;65(9):519-526. doi: 10.1007/s11748-017-0799-4. Epub 2017 Jun 28. PMID: 28660409.

Mordant P, Pricopi C, Legras A, Arame A, Foucault C, Dujon A, Le Pimpec-Barthes F, Riquet M. Prognostic factors after surgical resection of N1 non-small cell lung cancer. Eur J Surg Oncol. 2015 May;41(5):696-701. doi: 10.1016/j.ejso.2014.10.003. Epub 2014 Oct 15. PMID: 25454825.

Mynard N, Nasar A, Rahouma M, Lee B, Harrison S, Chow O, Villena-Vargas J, Altorki N, Port J. Extent of Resection Influences Survival in Early-Stage Lung Cancer With Occult Nodal Disease. Ann Thorac Surg. 2022 Sep;114(3):959-967. doi: 10.1016/j.athoracsur.2022.01.038. Epub 2022 Feb 15. PMID: 35181271.

Nakanishi R, Osaki T, Nakanishi K, Yoshino I, Yoshimatsu T, Watanabe H, Nakata H, Yasumoto K. Treatment strategy for patients with surgically discovered N2 stage IIIA non-small cell lung cancer. Ann Thorac Surg. 1997 Aug;64(2):342-8. doi: 10.1016/S0003- 4975(97)00535-3. PMID: 9262572.

Nakao M, Saji H, Mun M, Nakamura H, Okumura N, Tsuchida M, Sonobe M, Miyazaki T, Aokage K, Haruki T, Okada M, Suzuki K, Chida M. Prognostic Impact of Mediastinal Lymph Node Dissection in Octogenarians With Lung Cancer: JACS1303. Clin Lung Cancer. 2022 May;23(3):e176-e184. doi: 10.1016/j.cllc.2021.09.007. Epub 2021 Sep 24. PMID: 34690079.

Ni L, Lin G, Zhang Z, Sun D, Liu Z, Liu X. Surgery versus radiotherapy in octogenarians with stage Ia non-small cell lung cancer: propensity score matching analysis of the SEER database. BMC Pulm Med. 2022 Nov 10;22(1):411. doi: 10.1186/s12890-022-02177-7. PMID: 36357868; PMCID: PMC9650884.

Nomori H, Mori T, Izumi Y, Kohno M, Yoshimoto K, Suzuki M. Is completion lobectomy merited for unanticipated nodal metastases after radical segmentectomy for cT1 N0 M0/pN1-2 non-small cell lung cancer? J Thorac Cardiovasc Surg. 2012 Apr;143(4):820-4. doi: 10.1016/j.jtcvs.2011.10.045. Epub 2011 Nov 20. PMID: 22104683.

Okada M, Sakamoto T, Yuki T, Mimura T, Nitanda H, Miyoshi K, Tsubota N. Border between N1 and N2 stations in lung carcinoma: lessons from lymph node metastatic patterns of lower lobe tumors. J Thorac Cardiovasc Surg. 2005 Apr;129(4):825-30. doi: 10.1016/j.jtcvs.2004.06.016. PMID: 15821650.

Okada M, Tsubota N, Yoshimura M, Miyamoto Y, Nakai R. Evaluation of TMN classification for lung carcinoma with ipsilateral intrapulmonary metastasis. Ann Thorac Surg. 1999 Aug;68(2):326-30; discussion 331. doi: 10.1016/s0003-4975(99)00465-8. PMID: 10475390.

Okusanya OT, Lutfi W, Baker N, Dhupar R, Christie NA, Levy RM, Martinez-Meehan D, Siripong N, Luketich JD, Sarkaria IS. The association of robotic lobectomy volume and nodal upstaging in non-small cell lung cancer. J Robot Surg. 2020 Oct;14(5):709-715. doi: 10.1007/s11701-020-01044-z. Epub 2020 Jan 16. PMID: 31950332.

Oosterhuis JW, Theunissen PH, Bollen EC. Improved pre-operative mediastinal staging in non-small-cell lung cancer by serial sectioning and immunohistochemical staining of lymph-node biopsies. Eur J Cardiothorac Surg. 2001 Aug;20(2):335-8. doi: 10.1016/s1010- 7940(01)00777-1. PMID: 11463553.

Osaki T, Nagashima A, Yoshimatsu T, Tashima Y, Yasumoto K. Survival and characteristics of lymph node involvement in patients with N1 non-small cell lung cancer. Lung Cancer. 2004 Feb;43(2):151-7. doi: 10.1016/j.lungcan.2003.08.020. PMID: 14739035.

Prenzel KL, Mönig SP, Sinning JM, Baldus SE, Gutschow CA, Grass G, Schneider PM, Hölscher AH. Role of skip metastasis to mediastinal lymph nodes in non-small cell lung cancer. J Surg Oncol. 2003 Apr;82(4):256-60. doi: 10.1002/jso.10219. PMID: 12672010.

Razi SS, Nguyen D, Villamizar N. Lobectomy does not confer survival advantage over segmentectomy for non-small cell lung cancer with unsuspected nodal disease. J Thorac Cardiovasc Surg. 2020 Jun;159(6):2469-2483.e4. doi: 10.1016/j.jtcvs.2019.10.165. Epub 2019 Nov 21. PMID: 31928821.

Rea F, Marulli G, Callegaro D, Zuin A, Gobbi T, Loy M, Sartori F. Prognostic significance of main bronchial lymph nodes involvement in non-small cell lung carcinoma: N1 or N2? Lung Cancer. 2004 Aug;45(2):215-20. doi: 10.1016/j.lungcan.2004.01.017. PMID: 15246193.

Reichert M, Steiner D, Kerber S, Bender J, Pösentrup B, Hecker A, Bodner J. A standardized technique of systematic mediastinal lymph node dissection by video-assisted thoracoscopic surgery (VATS) leads to a high rate of nodal upstaging in early-stage non-small cell lung cancer. Surg Endosc. 2016 Mar;30(3):1119-25. doi: 10.1007/s00464-015-4312-9. Epub 2015 Jul 14. PMID: 26169635.

Riquet M, Assouad J, Bagan P, Foucault C, Le Pimpec Barthes F, Dujon A, Danel C. Skip mediastinal lymph node metastasis and lung cancer: a particular N2 subgroup with a better prognosis. Ann Thorac Surg. 2005 Jan;79(1):225-33. doi: 10.1016/j.athoracsur.2004.06.081. PMID: 15620948.

Riquet M, Manac'h D, Le Pimpec-Barthes F, Dujon A, Chehab A. Prognostic significance of surgical-pathologic N1 disease in non-small cell carcinoma of the lung. Ann Thorac Surg. 1999 Jun;67(6):1572-6. PMID: 10391257.

Riquet M, Manac'h D, Saab M, Le Pimpec-Barthes F, Dujon A, Debesse B. Factors determining survival in resected N2 lung cancer. Eur J Cardiothorac Surg. 1995;9(6):300-4. doi: 10.1016/s1010-7940(05)80186-1. PMID: 7546801.

Rocha AT, McCormack M, Montana G, Schreiber G. Association between lower lobe location and upstaging for early-stage non-small cell lung cancer. Chest. 2004 Apr;125(4):1424-30. doi: 10.1378/chest.125.4.1424. PMID: 15078755.

Sagawa M, Sakurada A, Fujimura S, Sato M, Takahashi S, Usuda K, Endo C, Aikawa H, Kondo T, Saito Y. Five-year survivors with resected pN2 nonsmall cell lung carcinoma. Cancer. 1999 Feb 15;85(4):864-8. doi: 10.1002/(sici)1097-0142(19990215)85:4<864::aid- cncr13>3.0.co;2-q. PMID: 10091763.

Sagawa M, Sato M, Sakurada A, Matsumura Y, Endo C, Handa M, Kondo T. A prospective trial of systematic nodal dissection for lung cancer by video-assisted thoracic surgery: can it be perfect? Ann Thorac Surg. 2002 Mar;73(3):900-4. doi: 10.1016/s0003-4975(01)03409-9. PMID: 11899198.

Sakamoto T, Tsubota N, Miyamoto Y, Yoshimura M. Analysis of lobectomy for small peripheral lung cancer supports extended segmentectomy. Jpn J Thorac Cardiovasc Surg. 1998 Apr;46(4):325-9. doi: 10.1007/BF03217749. PMID: 9619029.

Sakao Y, Miyamoto H, Yamazaki A, Oh T, Fukai R, Shiomi K, Saito Y. Prognostic significance of metastasis to the highest mediastinal lymph node in nonsmall cell lung cancer. Ann Thorac Surg. 2006 Jan;81(1):292-7. doi: 10.1016/j.athoracsur.2005.06.077. PMID: 16368383.

Sawyer TE, Bonner JA, Gould PM, Foote RL, Deschamps C, Trastek VF, Pairolero PC, Allen MS, Shaw EG, Marks RS, Frytak S, Lange CM, Li H. The impact of surgical adjuvant thoracic radiation therapy for patients with nonsmall cell lung carcinoma with ipsilateral mediastinal lymph node involvement. Cancer. 1997 Oct 15;80(8):1399-408. doi: 10.1002/(sici)1097-0142(19971015)80:8<1399::aid-cncr6>3.0.co;2-a. PMID: 9338463.

Sayan M, Satir Turk M, Celik A, Cuneyt Kurul I, Irfan Tastepe A. Surgical outcomes of early-stage small-cell lung cancer: single-center experience. Asian Cardiovasc Thorac Ann. 2019 Mar;27(3):187-191. doi: 10.1177/0218492319826724. Epub 2019 Jan 19. PMID: 30661378.

Sayar A, Turna A, Kiliçgün A, Solak O, Urer N, Gürses A. Prognostic significance of surgical-pathologic multiple-station N1 disease in non-small cell carcinoma of the lung. Eur J Cardiothorac Surg. 2004 Mar;25(3):434-8. doi: 10.1016/j.ejcts.2003.12.005. PMID: 15019674.

Seguin-Givelet A, Lutz J, Brian E, Grigoroiu M, Gossot D. Traitement chirurgical des cancers bronchiques non à petites cellules (CBNPC) de stade précoce par segmentectomie à thorax fermé : résultats préliminaires [Surgical treatment of early stage non-small cell lung cancer by thoracoscopic segmental resection]. Rev Mal Respir. 2018 May;35(5):521-530. French. doi: 10.1016/j.rmr.2018.02.004. Epub 2018 May 18. PMID: 29778621.

Sezen CB, Aksoy Y, Sonmezoglu Y, Citak N, Saydam O, Metin M. Prognostic factors for survival in patients with completely resected pN2 non-small-cell lung cancer. Acta Chir Belg. 2021 Feb;121(1):23-29. doi: 10.1080/00015458.2019.1658355. Epub 2019 Aug 29. PMID: 31437115.

Shibano T, Tsubochi H, Tetsuka K, Yamamoto S, Kanai Y, Minegishi K, Endo S. Left mediastinal node dissection after arterial ligament transection via video-assisted thoracoscopic surgery for potentially advanced stage I non-small cell lung cancer. J Thorac Dis. 2018 Dec;10(12):6458-6465. doi: 10.21037/jtd.2018.11.86. PMID: 30746188; PMCID: PMC6344703.

Shimada Y, Saji H, Kakihana M, Honda H, Usuda J, Kajiwara N, Ohira T, Ikeda N. Retrospective analysis of nodal spread patterns according to tumor location in pathological N2 non-small cell lung cancer. World J Surg. 2012 Dec;36(12):2865-71. doi: 10.1007/s00268- 012-1743-5. PMID: 22948194; PMCID: PMC3501158.

Shimada Y, Tsuboi M, Saji H, Miyajima K, Usuda J, Uchida O, Kajiwara N, Ohira T, Hirano T, Kato H, Ikeda N. The prognostic impact of main bronchial lymph node involvement in non-small cell lung carcinoma: suggestions for a modification of the staging system. Ann Thorac Surg. 2009 Nov;88(5):1583-8. doi: 10.1016/j.athoracsur.2009.04.065. PMID: 19853116.

Sun JM, Noh JM, Oh D, Kim HK, Lee SH, Choi YS, Pyo H, Ahn JS, Jung SH, Ahn YC, Kim J, Ahn MJ, Zo JI, Shim YM, Park K. Randomized Phase II Trial Comparing Chemoradiotherapy with Chemotherapy for Completely Resected Unsuspected N2-Positive Non- Small Cell Lung Cancer. J Thorac Oncol. 2017 Dec;12(12):1806-1813. doi: 10.1016/j.jtho.2017.09.1954. Epub 2017 Sep 28. PMID: 28962948.

Sura K, Grills IS, Vu CC, Stevens CW, Ye H, Guerrero TM. Improved Survival With Increased Time-To-Radiation and Sequential Chemotherapy After Surgery for pN2 Non-Small-cell Lung Cancer. Clin Lung Cancer. 2018 Mar;19(2):e185-e194. doi: 10.1016/j.cllc.2017.10.011. Epub 2017 Nov 20. PMID: 29158124.

Tachi R, Hattori A, Matsunaga T, Takamochi K, Oh S, Suzuki K. The impact on the prognosis of unsuspected N2 disease in non-small-cell lung cancer: indications for thorough mediastinal staging in the modern era. Surg Today. 2017 Jan;47(1):20-26. doi: 10.1007/s00595-016- 1372-y. Epub 2016 Jul 21. PMID: 27444026.

Takizawa T, Terashima M, Koike T, Akamatsu H, Kurita Y, Yokoyama A. Mediastinal lymph node metastasis in patients with clinical stage I peripheral non-small-cell lung cancer. J Thorac Cardiovasc Surg. 1997 Feb;113(2):248-52. doi: 10.1016/S0022-5223(97)70320-9. PMID: 9040617.

Tanaka F, Yanagihara K, Otake Y, Kawano Y, Miyahara R, Takenaka K, Katakura H, Ishikawa S, Ito H, Wada H. Prognostic factors in resected pathologic (p-) stage IIIA-N2, non-small-cell lung cancer. Ann Surg Oncol. 2004 Jun;11(6):612-8. doi: 10.1245/ASO.2004.07.013. Epub 2004 May 18. PMID: 15150069.

Thakur B, Yonghui D, Devkota M, Poudel B, Baral P. Surgical Results of Non-small Cell Lung Cancer in Nepal. JNMA J Nepal Med Assoc. 2014 Oct-Dec;52(196):992-6. PMID: 26982898.

Thomas DC, Arnold BN, Rosen JE, Salazar MC, Detterbeck FC, Blasberg JD, Boffa DJ, Kim AW. The Significance of Upfront Knowledge of N2 Disease in Non-small Cell Lung Cancer. World J Surg. 2018 Jan;42(1):161-171. doi: 10.1007/s00268-017-4165-6. PMID: 28799084.

Thomas PA, Couderc AL, Boulate D, Greillier L, Charvet A, Brioude G, Trousse D, D'Journo XB, Barlesi F, Loundou A. Early-stage non- small cell lung cancer beyond life expectancy: Still not too old for surgery? Lung Cancer. 2021 Feb;152:86-93. doi: 10.1016/j.lungcan.2020.12.009. Epub 2020 Dec 14. PMID: 33360807.

Turna A, Solak O, Kilicgun A, Metin M, Sayar A, Gürses A. Is lobe-specific lymph node dissection appropriate in lung cancer patients undergoing routine mediastinoscopy? Thorac Cardiovasc Surg. 2007 Mar;55(2):112-9. doi: 10.1055/s-2006-924626. PMID: 17377865.

Udelsman BV, Chang DC, Boffa DJ, Gaissert HA. Association of Lymph Node Sampling and Clinical Volume in Lobectomy for Non-Small Cell Lung Cancer. Ann Thorac Surg. 2023 Jan;115(1):166-173. doi: 10.1016/j.athoracsur.2022.05.051. Epub 2022 Jun 22. PMID: 35752354.

Ueda K, Kaneda Y, Saeki K, Fujita N, Zempo N, Esato K. Hilar lymph nodes in N2 disease: survival analysis of patients with non-small cell lung cancers and regional lymph node metastasis. Surg Today. 2002;32(4):300-4. doi: 10.1007/s005950200042. PMID: 12027193.

Van Klaveren RJ, Festen J, Otten HJ, Cox AL, de Graaf R, Lacquet LK. Prognosis of unsuspected but completely resectable N2 non-small cell lung cancer. Ann Thorac Surg. 1993 Aug;56(2):300-4. doi: 10.1016/0003-4975(93)91164-i. PMID: 8394066.

Vazirani J, Moraes J, Barnett S, Johnson DF, Knight S, Miller A, Wright G, Alam NZ, Conron M, Irving LB, Antippa P, Steinfort DP. Outcomes following resection of non-small cell lung cancer in octogenarians. ANZ J Surg. 2018 Dec;88(12):1322-1327. doi: 10.1111/ans.14861. Epub 2018 Oct 2. PMID: 30277303.

Wang R, Deng HY, Zhou J, Jiang R, Zhou Q. Surgical Consideration Based on Lymph Nodes Spread Patterns in Patients with Peripheral Right Middle Non-small Cell Lung Cancer 3 cm or Less. World J Surg. 2020 Oct;44(10):3530-3536. doi: 10.1007/s00268-020-05647-3. PMID: 32548710.

Wang S, Zhou W, Zhang H, Zhao M, Chen X. Analysis of predictive factors for postoperative survival for non small cell lung carcinoma patients with unexpected mediastinal lymph nodes metastasis. Thorac Cardiovasc Surg. 2014 Mar;62(2):126-32. doi: 10.1055/s-0033- 1338132. Epub 2013 Apr 12. PMID: 23585223.

Wang S, Zhou W, Zhang H, Zhao M, Chen X. Feasibility and long-term efficacy of video-assisted thoracic surgery for unexpected pathologic N2 disease in non-small cell lung cancer. Ann Thorac Med. 2013 Jul;8(3):170-5. doi: 10.4103/1817-1737.114291. PMID: 23922613; PMCID: PMC3731860.

Wang X, Yan S, Phan K, Yan TD, Zhang L, Yang Y, Wu N. Mediastinal lymphadenectomy fulfilling NCCN criteria may improve the outcome of clinical N0-1 and pathological N2 non-small cell lung

cancer. J Thorac Dis. 2016 Mar;8(3):342-9. doi: 10.21037/jtd.2016.02.49. PMID: 27076928; PMCID: PMC4805842.

Watanabe A, Mishina T, Ohori S, Koyanagi T, Nakashima S, Mawatari T, Kurimoto Y, Higami T. Is video-assisted thoracoscopic surgery a feasible approach for clinical N0 and postoperatively pathological N2 non-small cell lung cancer? Eur J Cardiothorac Surg. 2008 May;33(5):812-8. doi: 10.1016/j.ejcts.2008.01.064. Epub 2008 Mar 14. PMID: 18342533.

Wei WD, Wen ZS, Su XD, Lin P, Rong TH, Chen LK. [Multivariate survival analysis of 899 patients with non-small cell lung cancer after complete resection]. Ai Zheng. 2007 Nov;26(11):1231-6. Chinese. PMID: 17991324.

Wo Y, Li H, Zhang Y, Peng Y, Wu Z, Liu P, Shang Y, Hu H, Zhang Y, Xiang J, Sun Y. The impact of station 4L lymph node dissection on short-term and long-term outcomes in non-small cell lung cancer. Lung Cancer. 2022 Aug;170:141-147. doi: 10.1016/j.lungcan.2022.06.018. Epub 2022 Jun 29. PMID: 35780588.

Wu Yl, Huang ZF, Wang SY, Yang XN, Ou W. A randomized trial of systematic nodal dissection in resectable non-small cell lung cancer. Lung Cancer. 2002 Apr;36(1):1-6. doi: 10.1016/s0169-5002(01)00445-7. PMID: 11891025.

Xu F, Qi L, Yue D, Wang C. The effect of the extent of lymph node dissection for stage IA non-small-cell lung cancer on patient disease- free survival. Clin Lung Cancer. 2013 Mar;14(2):181-7. doi: 10.1016/j.cllc.2012.09.002. Epub 2012 Nov 27. PMID: 23195117.

Xu L, Su H, She Y, Dai C, Zhao M, Gao J, Xie H, Ren Y, Xie D, Chen C. Which N Descriptor Is More Predictive of Prognosis in Resected Non-small Cell Lung Cancer: The Number of Involved Nodal Stations or the Location-Based Pathological N Stage? Chest. 2021 Jun;159(6):2458-2469. doi: 10.1016/j.chest.2020.12.012. Epub 2020 Dec 19. PMID: 33352193.

Yang CF, Kumar A, Gulack BC, Mulvihill MS, Hartwig MG, Wang X, D'Amico TA, Berry MF. Long-term outcomes after lobectomy for non-small cell lung cancer when unsuspected pN2 disease is found: A National Cancer Data Base analysis. J Thorac Cardiovasc Surg. 2016 May;151(5):1380-8. doi: 10.1016/j.jtcvs.2015.12.028. Epub 2015 Dec 21. Erratum in: J Thorac Cardiovasc Surg. 2019 Jul;158(1):323. PMID: 26874598; PMCID: PMC4834248.

Yano T, Fukuyama Y, Yokoyama H, Kuninaka S, Terazaki Y, Uehara T, Asoh H, Ichinose Y. Long-term survivors with pN2 non-small cell lung cancer after a complete resection with a systematic mediastinal node dissection. Eur J Cardiothorac Surg. 1998 Aug;14(2):152-5. doi: 10.1016/s1010-7940(98)00162-6. PMID: 9755000.

Yildizeli B, Fadel E, Mussot S, Fabre D, Chataigner O, Dartevelle PG. Morbidity, mortality, and long-term survival after sleeve lobectomy for non-small cell lung cancer. Eur J Cardiothorac Surg. 2007 Jan;31(1):95-102. doi: 10.1016/j.ejcts.2006.10.031. Epub 2006 Nov 28. PMID: 17126556.

Yu DP, Bai LQ, Xu SF, Han M, Wang ZT. [Impact of TNM staging and treatment mode on the prognosis of non-small cell lung cancer]. Zhonghua Zhong Liu Za Zhi. 2009 Jun;31(6):465-8. Chinese. PMID: 19950561.

Yuan C, Tao X, Zheng D, Pan Y, Ye T, Hu H, Xiang J, Zhang Y, Chen H, Sun Y. The lymph node status and histologic subtypes influenced the effect of postoperative radiotherapy on patients with N2 positive IIIA non-small cell lung cancer. J Surg Oncol. 2019 Mar;119(3):379- 387. doi: 10.1002/jso.25308. Epub 2018 Dec 9. PMID: 30536966.

Zhang D, Chen X, Zhu D, Qin C, Dong J, Qiu X, Fan M, Zhuo Q, Tang X. Intrapulmonary lymph node metastasis is common in clinically staged IA adenocarcinoma of the lung. Thorac Cancer. 2019 Feb;10(2):123-127. doi: 10.1111/1759-7714.12908. Epub 2018 Nov 23. PMID: 30468025; PMCID: PMC6360232.

Zhang S, Wang L, Lu F, Pei Y, Yang Y. [Correlation between Lymph Node Ratio and Clinicopathological Features and Prognosis of IIIa- N2 Non-small Cell Lung Cancer]. Zhongguo Fei Ai Za Zhi. 2019 Nov 20;22(11):702-708. Chinese. doi: 10.3779/j.issn.1009- 3419.2019.11.04. PMID: 31771739; PMCID: PMC6885420.

Zhang SY, Wang X, Zhu ZH, Zeng CG, Rong TH, Zheng L, Fu JH, Xie ZM, Zhang X, Yu H. [Clinical value of cervical mediastinoscopy in non-small cell lung cancer of stage I]. Ai Zheng. 2005 Mar;24(3):349-52. Chinese. PMID: 15757540.

Zhang Y, Chen C, Hu J, Han Y, Huang M, Xiang J, Li H. Early outcomes of robotic versus thoracoscopic segmentectomy for early-stage lung cancer: A multi-institutional propensity score-matched analysis. J Thorac Cardiovasc Surg. 2020 Nov;160(5):1363-1372. doi: 10.1016/j.jtcvs.2019.12.112. Epub 2020 Jan 25. PMID: 32113718.

Zhang Z, Miao J, Chen Q, Fu Y, Li H, Hu B. Assessment of non-lobe-specific lymph node metastasis in clinical stage IA non-small cell lung cancer. Thorac Cancer. 2019 Jul;10(7):1597-1604. doi: 10.1111/1759-7714.13121. Epub 2019 Jun 17. PMID: 31206253; PMCID: PMC6610282.

Zhao F, Zhen FX, Zhou Y, Huang CJ, Yu Y, Li J, Li QF, Zhu CX, Yang XY, You SH, Wu QG, Qin XY, Liu Y, Chen L, Wang W. Clinicopathologic predictors of metastasis of different regional lymph nodes in patients intraoperatively diagnosed with stage-I non-small cell lung cancer. BMC Cancer. 2019 May 14;19(1):444. doi: 10.1186/s12885-019-5632-2. PMID: 31088404; PMCID: PMC6518627.

Zhao J, Li W, Wang M, Liu L, Fu X, Li Y, Xu L, Liu Y, Zhao H, Hu J, Liu D, Shen J, Yang H, Li X. Video-assisted thoracoscopic surgery lobectomy might be a feasible alternative for surgically resectable pathological N2 non-small cell lung cancer patients. Thorac Cancer. 2021 Jan;12(1):21-29. doi: 10.1111/1759-7714.13680. Epub 2020 Nov 18. PMID: 33205914; PMCID: PMC7779187.

Zhao Y, Mao Y, He J, Gao S, Zhang Z, Ding N, Xue Q, Gao Y, Wang D, Zhao J, Tan F, Yuan L, Li F, Wang S, Yang L. Lobe-specific Lymph Node Dissection in Clinical Stage IA Solid-dominant Non-small-cell Lung Cancer: A Propensity Score Matching Study. Clin Lung Cancer. 2021 Mar;22(2):e201-e210. doi: 10.1016/j.cllc.2020.09.012. Epub 2020 Oct 15. PMID: 33187913.

Zhong C, Yao F, Zhao H. Clinical outcomes of thoracoscopic lobectomy for patients with clinical N0 and pathologic N2 non-small cell lung cancer. Ann Thorac Surg. 2013 Mar;95(3):987-92. doi: 10.1016/j.athoracsur.2012.10.083. Epub 2012 Dec 20. PMID: 23261117.

Zhou H, Tapias LF, Gaissert HA, Muniappan A, Wright CD, Wain JC, Donahue DM, Morse CR, Mathisen DJ, Lanuti M. Lymph Node Assessment and Impact on Survival in Video-Assisted Thoracoscopic Lobectomy or Segmentectomy. Ann Thorac Surg. 2015 Sep;100(3):910-6. doi: 10.1016/j.athoracsur.2015.04.034. Epub 2015 Jul 10. PMID: 26165483.

Zhou W, Chen X, Zhang H, Zhang H, Zhao M. Video-assisted thoracic surgery lobectomy for unexpected pathologic N2 non-small cell lung cancer. Thorac Cancer. 2013 Aug;4(3):287-294. doi: 10.1111/1759-7714.12015. PMID: 28920251.

Excluded papers based on duplicate or overlapping cohort

Citak N, Sayar A, Metin M, Büyükkale S, Kök A, Solak O, Yurt S, Gürses A. The Prognostic Significance of Metastasis to Lymph Nodes in Aortopulmonary Zone (Stations 5 and 6) in Completely Resected Left Upper Lobe Tumors. Thorac Cardiovasc Surg. 2015 Oct;63(7):568- 76. doi: 10.1055/s-0035-1546463. Epub 2015 Apr 20. PMID: 25893919.

Sun, J., Ahn, M., Ahn, J., Kim, H., Ahn, Y., Zo, J., Shim, Y. and Park, K., 2017. Randomized phase ii trial comparing chemoradiotherapy with chemotherapy for completely resected unsuspected N2-positive non-small cell lung cancer. Annals of Oncology, 28, p.v457.

Excluded papers based on irretrievable or unavailable papers

Carbognani P, Rusca M, Spaggiari L, Cattelani L, Bobbio A, Romani A, Solli P. Mediastinoscopy, thoracoscopy and left anterior mediastinotomy in the diagnosis of N2 non small cell lung cancer. J Cardiovasc Surg (Torino). 1996 Dec;37(6 Suppl 1):177-8. PMID: 10064372.

Dalton R, Keller S. Survival following resection of clinically occult N2 non small cell lung cancer. J Cardiovasc Surg (Torino). 1994 Dec;35(6 Suppl 1):13-7. PMID: 7775526.

Dell'amore A, Monteverde M, Caroli G, Sanna S, Stella F, Bini A. Surgical results and survival of older patients with unsuspected N_2 (stage IIIA) non-small cell lung cancer. G Chir. 2013 Mar;34(3):53-8. PMID: 23578406.

Niizeki H, Morikawa T, Okushiba S, Kondo S, Katoh H. Survival and prognostic factors in resected cN2-pN0 non-small cell lung cancer. Ann Thorac Cardiovasc Surg. 2004 Feb;10(1):9-13. PMID: 15008692.

Shiba M, Yamaguchi Y, Takeda T. [Incidence of lymph node involvement in pN2 non-small cell lung carcinoma and reevaluation of the lymph node dissection method]. Kyobu Geka. 1994 Jan;47(1):33-6. Japanese. PMID: 8277629.
